# Validity of the formative physical therapy Student and Clinical Instructor Performance Assessment Instrument in the United States: a quasi-experimental, time-series study

**DOI:** 10.3352/jeehp.2025.22.26

**Published:** 2025-09-26

**Authors:** Sean Gallivan, Jamie Bayliss

**Affiliations:** 1 Department of Physical Therapy, University of Dayton, Dayton, OH, USA; 2 Department of Physical Therapy, Mount Saint Joseph University, Cincinnati, OH, USA; The Catholic University of Korea, Korea

**Keywords:** Counselling, Educational status, Physical therapists, Students, United States

## Abstract

**Purpose:**

The aim of this study was to assess the validity of the Student and Clinical Instructor Performance Assessment Instrument (SCIPAI), a novel formative tool used in physical therapist education to assess student and clinical instructor (CI) performance throughout clinical education experiences (CEEs). The researchers hypothesized that the SCIPAI would demonstrate concurrent, predictive, and construct validity while offering additional contemporary validity evidence.

**Methods:**

This quasi-experimental, time-series study had 810 student-CI pairs complete 2 SCIPAIs before and after CEE midpoint, and an endpoint Clinical Performance Instrument (CPI) during beginning to terminal CEEs in a 1-year period. Spearman rank correlation analyses used final SCIPAI and CPI like-item scores to assess concurrent validity; and earlier SCIPAI and final CPI like-item scores to assess predictive validity. Construct validity was assessed via progression of student and CI performance scores within CEEs using Wilcoxon signed-rank testing. No randomization/grouping of subjects occurred.

**Results:**

Moderate correlation existed between like final SCIPAI and CPI items (P<0.005) and between some like items of earlier SCIPAIs and final CPIs (P<0.005). Student performance scores demonstrated progress from SCIPAIs 1 to 4 within CEEs (P<0.005). While a greater number of CIs demonstrated progression rather than regression in performance from SCIPAI 1 to SCIPAI 4, the greater magnitude of decreases in CI performance contributed to an aggregate ratings decrease of CI performance (P<0.005).

**Conclusion:**

The SCIPAI demonstrates concurrent, predictive, and construct validity when used by students and CIs to rate student performance at regular points throughout clinical education experiences.

## Graphical abstract

[Fig f3-jeehp-22-26]

## Introduction

### Background/rationale

Physical therapist education programs (PTEPs) must use acceptable practices to assess student and instructor performance in clinical settings to manage the quality of the educational product and meet accreditation standards [1]. Clinical education experience (CEE) performance assessment processes of PTEPs are supported by the use of standardized, nationally-utilized student performance assessment tools [2-4] and clinical instructor (CI) performance assessment tools [5,6]. These tools are used summatively at the endpoint of a CEE to assess either student or CI performance, and typically offer opportunities for formative use at the midpoint of CEEs.

As length of summative assessments limits how often students and CIs are asked to complete them during a CEE, shorter formative weekly feedback forms are used by PTEPs to augment summative student performance assessment. Formative assessment identifies prevailing performer strengths and weaknesses, allowing students and instructors time to improve performance prior to final, high-stakes summative assessment. Contemporary assessment literature promotes the careful combination of formative and summative assessments to strengthen the benefits of the assessment [7,8]. While providing summative assessments, the physical therapy profession is lacking a complementary, formative tool that offers contemporary validity evidence when used for assessment of student and CI performance throughout a CEE. Further, the literature does not detail the how CI performance changes throughout a CEE.

To fill this gap in validity evidence for formative assessment in physical therapist (PT) clinical education and improve the quality of teaching and learning in CEEs, a novel formative tool, the Student and Clinical Instructor Performance Assessment Instrument (SCIPAI), has been developed for student and CI development. The SCIPAI assesses both student and CI performance at multiple points throughout a CEE, using a formative, low-stakes approach to complement current summative tools. The tool embraces self- and other-assessment concepts [9], and purposefully limits the number of items assessed for feasible, regular use. This study provides the healthcare education assessment community with its first example of contemporary validity evidence for use of a formative weekly feedback assessment for physical therapy CEEs, an example of the use of complementary formative-summative assessments for the improvement of clinic-based teaching and learning, and reinforcement of the shift in validity assessment from validity of a tool (classical) to validity evidence of the intended use of a tool (contemporary) [10,11].

Contemporary validity theory [10-12] connects the intended use of an assessment with its impact (e.g., formative use [low-stakes impact and performance adjustment] and summative use [high-stakes, pass/fail determinations]) by considering 6 categories of validity evidence: content, response process, internal structure, relational, consequences, and generalizability/reliability [12].

### Objectives

The aim of this multi-institutional study was to offer a brief review of contemporary validity evidence for use of the SCIPAI while offering a more detailed analysis of 3 aspects of the relational category of validity evidence: construct validity through progression of student and CI performance ratings within CEEs; concurrent validity of SCIPAI student performance ratings when correlated with an established, valid tool, the Clinical Performance Instrument [2] (CPI); and predictive validity of earlier SCIPAI student performance ratings with final CPI scores. The researchers proposed several hypotheses about the relational validity evidence for this study’s use of the SCIPAI: (1) student SCIPAI performance scores would increase throughout a CEE; (2) CI SCIPAI performance scores would increase throughout a CEE; (3) student final SCIPAI scores would correlate with like-item scores on the final CPI; and (4) student SCIPAI scores on earlier SCIPAIs would correlate with like-item scores on the final CPI.

## Methods

### Ethics statement

The University of Dayton (No. 043021) and Western Kentucky University (No. 22016) Institutional Review Boards approved this study as exempt human subjects research. Potential participants from participating PTEPs received the study’s invitation to participate and could opt out of their deidentified data being included in the study. Researchers only had access to deidentified participant data.

### Study design

A quasi-experimental, time-series study design was used for the psychometric assessment (construct, concurrent, and predictive validity) of the SCIPAI.

### Setting

The researchers invited all 14 accredited PTEPs located in Ohio and Kentucky, USA, to enroll all calendar-year 2021 CEEs in this study.

### Participants

All 14 Ohio and Kentucky PTEPs were offered SCIPAI access for each CEE in 2021. A non-probability convenience sample of student-CI pairs from the 8 Ohio and Kentucky PTEPs using the SCIPAI was invited to participate. Students completing full-time CEEs of 6 to 12 weeks were eligible to participate. To minimize variability in clinical learning approaches, all participants used a 1:1 student:CI model of clinical learning. Students using a model that included more than 1 CI or student were excluded from the study. The lack of evidence to support CI demographics (e.g., years as clinician, years as clinical instructor, training as a credentialed clinical instructor) as impacting student outcomes [13] and evidence of significant variability in CI demographics [14] suggested that minimizing variations of the study sample from the overall CI population could best be achieved by maximizing CI sample size.

### Interventions

**Fig. 1.**
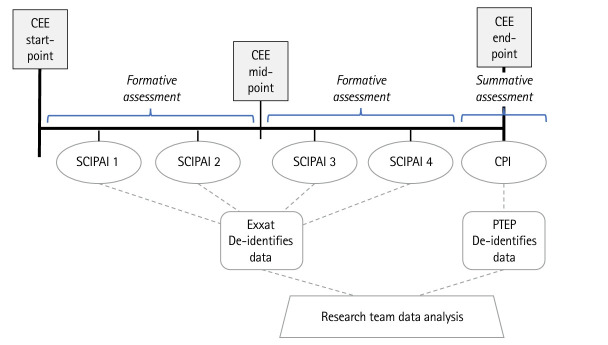
Assessment protocol for each clinical education experience (CEE). SCIPAI, Student and Clinical Instructor Performance Assessment Instrument; CPI, Clinical Performance Instrument.

Students and CIs used Exxat, a physical therapy education management platform, to complete at least 4 SCIPAIs per CEE, including 2 SCIPAIs prior to the midpoint and 2 SCIPAIs after the midpoint of each CEE (Fig. 1). Subjects also completed the APTA CPI version 2006 at the endpoint of the CEE, using the CPI’s Liaison International assessment platform. Student and CI training videos informed participants of the SCIPAI’s purpose, rating scale, and completion processes. Participating PTEPs authorized Exxat to provide the researchers with de-identified SCIPAI data after each CEE. Programs provided the researchers with de-identified CPI data.

### Data sources/measurement

The SCIPAI is a formative tool that uses a 0–100 rating scale (higher scores indicate better performance) to rate different areas of student and CI performance at regular points throughout a CEE (Supplement 1). Students assess and CIs self-assess CI performance in the areas of supervision and feedback. Students self-assess and CIs assess student performance in the areas of evaluation, treatment, communication, and professional behavior. Raters provide comments in support of item ratings, performer accomplishments for the past week, and goals for the next week. Finally, raters identify the percentage of the caseload the student is managing independently. Student and CI complete the tool independently online before discussing completed SCIPAIs in person. The SCIPAI demonstrated concurrent validity and construct validity in a single-institution pilot study [15].

The APTA CPI version 2006 is a summative tool that assesses 18 student clinical performance items, including communication, professional behavior, evaluation, examination, procedural interventions, and educational interventions, using a 21-point scale (Supplement 2). Students self-assess and CIs assess student performance while providing comments supportive of ratings. A systematic review of 14 student clinical performance assessment tools identified the CPI as the only tool demonstrating the study’s construct validity standard amongst tools commonly used by PTEPs in the United States [16]. Just prior to the initiation of this study, 264 PTEPs and 51,287 clinical sites were utilizing the CPI [17].

### Outcome variables

This study assessed the SCIPAI’s concurrent, predictive, and construct validity of its student performance variables: evaluation, treatment, communication, and professional behavior; and the CPI’s communication, professional behavior, evaluation, examination, procedural interventions, and educational interventions variables. The SCIPAI’s CI performance variables of supervision and feedback were used to assess the construct validity of the CI component of the SCIPAI

### Bias

To minimize bias, the researchers only had access to deidentified participant data and used measurement tools that had been previously validated either by pilot study [15] (SCIPAI) or larger, multi-institutional study [2] (CPI). Concurrent and predictive validity of the CI performance assessment was not conducted due to the literature identifying no statistically valid student assessment of CI performance.

### Study size

SPSS power analyses estimated minimum sample sizes. Researchers used the common statistical power of 0.80 and minimum effect sizes (0.30 for correlations, 0.20 for means comparisons) so as to maximize the required least number of subjects, yielding sample minimums of 89 subjects for correlations and 199 for means comparisons.

### Assignment method

No grouping of subjects occurred for this single-group time-series study.

### Statistical methods

Concurrent validity was assessed using Spearman rank correlation analyses of student performance items and sum of scores values of the SCIPAI with like-items of a “gold-standard”. CPI version 2006 was identified as a “gold standard” for concurrence assessment due to its high ranking in the literature for construct validity [16] and its majority use amongst United States PTEPs [17]. Predictive validity was assessed using Spearman rank correlation of sums of scores of earlier SCIPAIs and final CPIs. Cohen guidelines for correlational effect sizes (Cohen’s r: small 0.10–<0.30, medium 0.30–<0.50, large ≥0.50) informed results interpretation [18,19]. Construct validity was assessed via progression of student and CI performance scores from SCIPAI 1 to SCIPAI 4 within a CEE using Wilcoxon signed-rank testing. A P-value of 0.05 provided the threshold for determining statistical significance.

The researcher conducted all statistical analyses using IBM SPSS Statistics for Windows version 23.0 (IBM Corp.).

## Results

### Participants

**Fig. 2.**
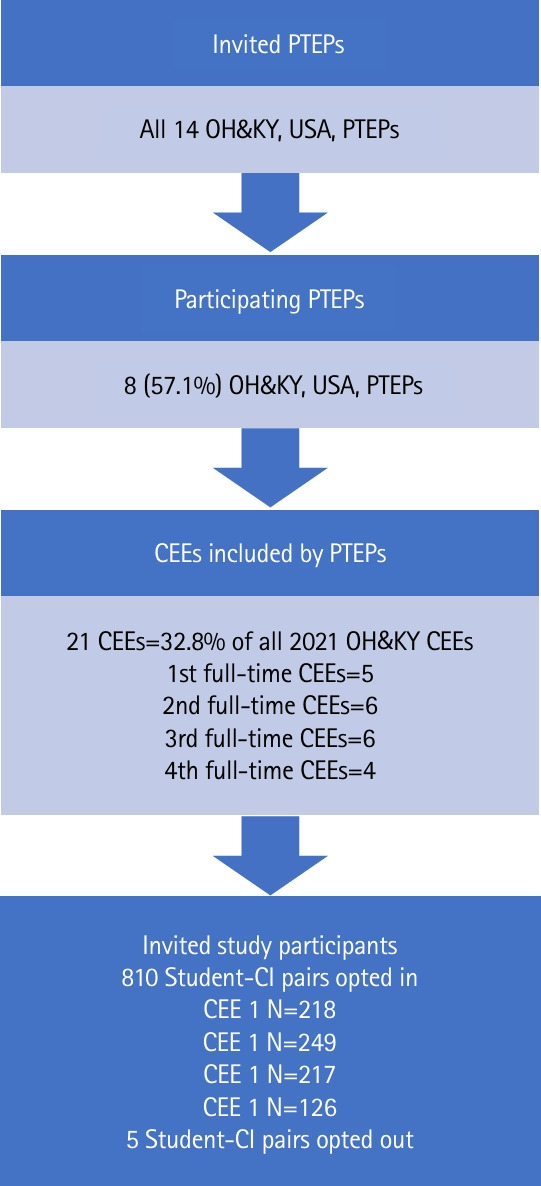
Participants. PTEPS, Physical Therapy Education Programs; OH&KY, Ohio & Kentucky; CEEs, clinical education experiences; CI, clinical instructor.

Results were generated using the study raw data (Dataset 1). A non-probability convenience sample of 811 student and CI pairs from 8 PTEPs (57.1%) participated in the study, completing a combined 21 CEEs (32.8% of CEEs of all 14 Ohio and Kentucky PTEPs). Clinical instructors were licensed PTs in various clinical settings. That 810 of 815 (99.4%) CIs involved in 1:1 model of clinical instruction from 8 of 14 (57.1%) Ohio and Kentucky PTEPs participated in this study lessens the chance that variations from the literature “norm” of widely varying CI population demographics occurred. Distribution of students across level of clinical instructors is illustrated in Fig. 2.

### Contemporary validity categorical evidence

A summary of the validity evidence for each category of contemporary validity evidence of this study’s use of the SCIPAI is presented in Table 1.

### Relations with other variables validity evidence

#### Concurrent validity

Spearman rank correlation analyses exploring the relationship of student performance items of the final SCIPAI to like-items of the final CPI showed moderate correlation between most like-items (P<0.01) (Table 2). For CI assessment of student performance, evaluation, treatment, and communication each had medium correlation between ratings on similar final SCIPAI and CPI items (r_s_=0.305–0.386, P<0.01); professional behavior had low correlation (r=0.203, P<0.01). For student self-assessment: evaluation (examination) and treatment each had a medium correlation between ratings on similar final SCIPAI and CPI items (r_s_=0.300–0.404, P<0.01); evaluation (evaluation), professional behavior, and communication had low correlation (r_s_=0.137–0.293, P<0.01). CI and student ratings of student performance on the SCIPAI treatment item demonstrated the strongest correlations with its CPI like-item, while their ratings of student professional behavior and communication demonstrated the weakest like-item correlations.

#### Predictive validity

Table 2 also illustrates the exploration of the relationship between sums of scores on earlier SCIPAIs and final CPI sums of scores. When rated by CIs, SCIPAI #2 (r=0.331, P<0.01) and SCIPAI #3 (r=0.334, P<0.01) demonstrate medium correlations with final CPI sums of scores; whereas, SCIPAI #1 demonstrates low correlation (r=0.275, P<0.01) with final CPI sums of scores. When rated by students, each of SCIPAI #1–3 demonstrate low correlations with final CPI sums of scores.

#### Construct validity

Clinical instructor performance scores within CEEs (Table 3), when assessed by CIs, increased from SCIPAI 1 to SCIPAI 4 for feedback and decreased for supervision (P<0.05). When rated by students, CI performance scores decreased from SCIPAI 1 to SCIPAI 4 for feedback and showed no significant change in supervision.

While only 1 of 4 Wilcoxon signed rank analyses (CI ratings of CI feedback) yielded a significant positive change in aggregate CI performance, all 4 analyses demonstrated a higher frequency of positive changes than negative changes in individual CI performance by an average ratio of 2.3:1 (Table 4).

Table 3 also illustrates the progression of student performance scores within CEEs. Student performance, as rated by students and CIs, increased from SCIPAI 1 to SCIPAI 4 within CEEs for all items (P<0.05).

## Discussion

### Key results

This multi-institutional study assessed the validity of the use of the SCIPAI as a formative, self and other, CI and student performance assessment for use at multiple points throughout CEEs, proposing 4 validity hypotheses assessing 3 types of relationships with other variables validity evidence (Table 5). Each hypothesis regarding student performance measures was supported by the study results and contributes to the SCIPAI as demonstrating contemporary validity [10-12].

Concurrent and predictive validity results were of moderate strength, with CI ratings of student performance demonstrating stronger correlations than student self-ratings. While construct validity assessment of CI and student ratings of student performance demonstrated significant gains (P-values all <0.001) from earlier to later points within CEEs, CI performance only demonstrated significant gain in CI self-assessment of feedback.

While this study focused on relationships with other variables validity evidence, this study supports the SCIPAI as demonstrating evidence for all six categories of Messick’s contemporary validity framework [12] (Table 1). An analysis of the SCIPAI’s internal structure is presented in Supplement 3.

### Interpretation

The results support the use of the SCIPAI as a formative, self and other, student performance assessment for use at multiple points throughout CEEs. Moderate strength correlations with CPI provide confidence that the student performance items of the SCIPAI align with the student performance items of the CPI, allowing for multiple points of student adjustment of clinical performance items that will be assessed on the final CPI. Comparisons of student SCIPAI ratings at multiple points within CEEs demonstrate a progression of scores, suggesting a call to action when SCIPAI scores are not progressing. The sum of student performance results suggests the formative SCIPAI can be used at multiple points throughout clinicals in a complementary manner with the CPI’s summative, end-point assessment.

While Wilcoxon signed rank test results show no significant change for student ratings of CI supervision, positive changes for CI ratings of CI feedback, and negative changes for CI ratings of CI supervision and student ratings of CI feedback, the results also show that positive changes in student and CI ratings of CI supervision and feedback occur at a greater frequency than negative performance changes by a ratio of greater than 2:1 (Table 4, Table 5).

Further analyses of CI performance illustrate that, for cases of change in individual CI supervision and feedback scores, changes will be positive in 69% of cases. Yet, this greater preponderance of positive changes in CI performance is overshadowed by the greater magnitude of cases of negative changes, yielding significant negative aggregate changes in student and CI ratings of CI feedback and no significant aggregate change in student ratings of CI supervision.

This significant minority of negative changes in CI ratings as the CEE progresses may suggest a need for students and CIs to better recognize the changing needs for supervision and feedback as student performance progresses throughout a CEE. As directors of clinical education (DCEs) and site coordinators of clinical education (SCCEs) maintain real-time access to completed SCIPAIs, the progression of score results supports DCE and SCCE awareness and potential intervention when student performance scores are not progressing or when CI performance scores are regressing during a CEE. Medium strength correlations of SCIPAI and CPI scores support DCE confidence that final CPI scores will be reflective of SCIPAI scores and take action when SCIPAI scores appear concerning.

### Comparison with previous studies

This study confirms the validity findings of the SCIPAI pilot study on student performance while calling into question the tool’s CI performance validity evidence [15]. It provides multiple validity assessments to align with the literature on contemporary validity [10-12]. It supports the contemporary validity principle of connecting the use of the tool (formative) with its consequence (performance improvement prior to final summative assessment via low-stakes interpretation of SCIPAI data) [8]. The SCIPAI provides multi-rater assessment to address the bias concerns of single-rater tools demonstrated in the literature [9]. This study’s validity evidence of the use of the SCIPAI addresses the current absence in the literature of a valid, formative tool intended for use throughout CEEs to aid student improvement while highlighting the opportunity to further explore how the SCIPAI can contribute to CI development. Finally, this tool offers to all healthcare professions an assessment approach that could help overcome documented resistance to other formative assessments [20].

### Limitations

This study was conducted on a small number of PTEPs in the same US region. Potential moderating factors of the duration of CEEs and more specific timing of SCIPAI measures were not analyzed.

### Generalizability

Contributing to this study’s generalizability is that it was conducted with students from public and private universities, urban and rural PTEPs, and students from earlier, middle, and later CEEs within varied PTEP curricula. Examining PTEPs across regions may provide further generalizability of research findings. The generalizability is further buoyed by the study exceeding by hundreds of cases the minimum number of cases needed for comparison and correlational analyses.

### Suggestions for further studies

While providing immediate practical implications and filling a gap in CEE performance assessment, this study provides robust opportunities for further research to meet prevailing needs in PT CE. This study can be replicated with the recently-released CPI 3.0 or other valid, summative tools. The SCIPAI could be further studied to determine performance ratings consistent with first, intermediate, and terminal CEEs. The regular, documented CI and student assessment of CI performance that the SCIPAI facilitates creates a culture shift that “normalizes” supervisee (student) assessment of a supervisor/instructor (CI) during a CEE. Each of these phenomena inspires ample opportunities to explore the SCIPAI impact on student-CI relationships, CI performance improvement, and their influence on student performance.

### Conclusion

This multi-institutional study provides contemporary validity evidence for use of the SCIPAI as a formative tool throughout CEEs. Its results support construct, concurrent, and predictive validity hypotheses for the assessment of student performance while offering content, response process, consequence, internal structure, and generalizability validity evidence. The physical therapy profession now has a valid, formative tool that provides for regular student self and other performance assessment throughout CEEs that can determine whether a student is progressing appropriately toward CEE performance objectives. Finally, the results highlight the need to further explore tools that assess how a CI’s teaching approach might need refining during CEEs.

## Figures and Tables

**Figure f3-jeehp-22-26:**
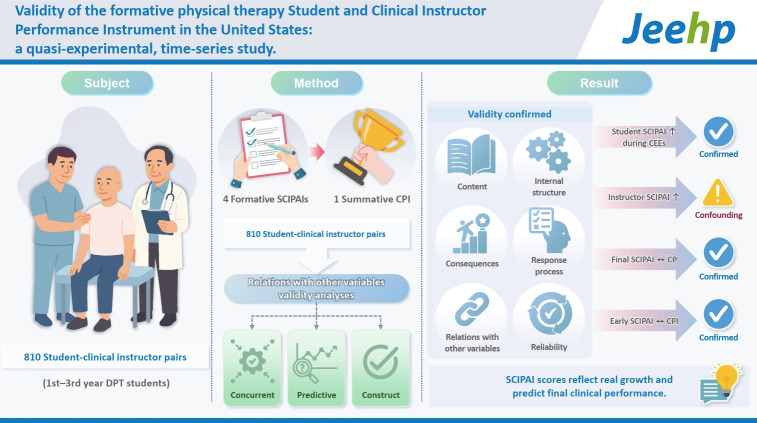


**Table 1.** Application of standards for educational and psychological testing and Messick’s framework of unitary validity to use of the SCIPAI

Validity evidence type	Meaning	SCIPAI evidence
Content	Content represents construct being assessed	Student performance content based upon prior instruments; CI performance content based upon accreditation standards; Student and CI performance items developed by credentialed CIs and reviewed by academic clinical educator content experts
Response process	Evidence of data integrity	Student and CI raters uniformly trained in use of instrument; simple, feasible scoring instructions and process; clean data entry & management
Internal structure	Relationships of items within the assessment tool (e.g., reliability, internal consistency)	Inter-item correlations, corrected item-total correlations, and split-half analyses all support acceptable internal structure reliability (See Supplement 3. Internal structure analyses)
Relations with other variables	Assessment item associations with variables external to the assessment (e.g., concurrent and predictive validity, time/group-based score differences)	Concurrent and predictive validity analyses; progression of scores analyses (see Tables 2–5)
Consequences	Impact on examinee, teaching, and learning	As a low-stakes, formative tool, the intention is for the use of the tool to increase communication to allow addressing of opportunities for improvement of teaching and learning. Comments on later SCIPAIs indicate addressing items highlighted on earlier SCIPAIs
Generalizability/reliability	Adequate number of cases, assessment encounters, raters	Power analysis for comparisons (N=199) and correlations (N=89) each exceeded by hundreds of cases, with N of raters equivalent to N of cases. Each rater/ratee pair had 4 assessment encounters

SCIPAI, Student and Clinical Instructor Performance Assessment Instrument; CI, clinical instructor.

**Table 2.** Concurrent and predictive validity assessments of SCIPAI and CPI student performance like items

Final SCIPAI item	Final CPI item	Clinical instructor as rater			Student as rater		
		No. of participants	Effect size^a)^	Cohen category^b)^	No. of participants	Effect size^a)^	Cohen category^b)^
Concurrent validity item pairs							
Evaluation	Examination	609	0.362	Medium	661	0.300	Medium
Evaluation	Evaluation	609	0.355	Medium	661	0.293	Small
Treatment	Plan of care	610	0.380	Medium	661	0.392	Medium
Treatment	Procedural intervention	610	0.386	Medium	661	0.404	Medium
Treatment	Educational intervention	610	0.372	Medium	661	0.401	Medium
Communication	Communication	608	0.305	Medium	660	0.285	Small
Professional behavior	Professional behavior	608	0.203	Small	659	0.137	Small
Sum of SCIPAI scores (0–400)	Sum of CPI scores^d)^ (18–378)	615	0.415	Medium	660	0.388	Medium
Predictive validity item pairs							
Sum of scores SCIPAI^c)^ #1 (0–400)	Sum of scores final CPI^d)^ (18–378)	630	0.275	Small	672	0.150	Small
Sum of scores SCIPAI^c)^ #2 (0–400)	Sum of scores final CPI^d)^ (18–378)	697	0.331	Medium	728	0.253	Small
Sum of scores SCIPAI^c)^ #3 (0–400)	Sum of scores final CPI^d)^ (18–378)	650	0.334	Medium	689	0.266	Small

SCIPAI, Student & Clinical Instructor Performance Assessment Instrument; CPI, Clinical Performance Instrument.

a) P-values for all analyses significant, P<0.01.

b) Cohen’s r guidelines for correlational effect sizes (small 0.10–<0.30; medium 0.30–<0.50; large ≥0.50).

c) Sum of 4 SCIPAI items each rated on a 0–100 scale; total possible sum 0–400.

d) Sum of 18 CPI items each rated on a 1–21 scale; total possible sum 18–378.

**Table 3.** Clinical instructor and student performance—progression of scores within clinical education experiences

Item	Rater	No. of participants	SCIPAI 1 mean score	SCIPAI 4 mean score	Z-score^a)^	P-value^a)^
CI performance						
Supervision	CI	478	91.6	90.1	3.105	0.002^*^
Supervision	Student	590	95.9	94.1	1.231	0.218
Feedback	CI	477	91.7	93.1	5.610	<0.001^*^
Feedback	Student	588	96.6	96.1	3.553	<0.001^*^
Student performance						
Evaluation	CI	506	52.8	86.6	18.393	<0.001^*^
Evaluation	Student	572	49.9	84.2	19.573	<0.001^*^
Treatment	CI	514	51.1	86.4	18.653	<0.001^*^
Treatment	Student	585	49.5	84.9	20.271	<0.001^*^
Communication	CI	518	79.4	94.4	14.447	<0.001^*^
Communication	Student	587	78.9	93.3	15.628	<0.001^*^
Professional behavior	CI	520	95.5	99.1	7.959	<0.001^*^
Professional behavior	Student	587	94.0	98.8	12.069	<0.001^*^
Sum of scores	CI	521	270.6	363.3	18.679	<0.001^*^
Sum of scores	Student	582	275.0	361.12	20.456	<0.001^*^

SCIPAI, Student & Clinical Instructor Performance Assessment Instrument; CI, clinical instructor.

* *P<0.05 (statistically significant).

a) Repeated measure Wilcoxon signed rank test.

**Table 4.** Clinical instructor performance—directionality of changes of scores within clinical education experiences

Item	Rater	No. of participants	CIs with positive changes SCIPAI 1–SCIPAI 4	CIs with no changes SCIPAI 1–SCIPAI 4	CIs with negative changes SCIPAI 1–SCIPAI 4
Supervision	CI	478	172	231	75
Supervision	Student	590	141	370	79
Feedback	CI	477	177	238	62
Feedback	Student	588	134	393	61
Total	All	2,133	624	1,232	277

CI, clinical instructor; SCIPAI, Student & Clinical Instructor Performance Assessment Instrument.

**Table 5.** Hypotheses assessment results

Hypothesis	Validity assessed	Statistical analysis	Result threshold	Hypothesis/validity outcome
Student SCIPAI scores will increase during CEEs.	Construct	Wilcoxon signed rank	P<0.05	Confirmed
CI SCIPAI scores will increase during CEEs.	Construct	Wilcoxon signed rank	P<0.05	Not confirmed
Student final SCIPAI scores will correlate with like-item final CPI scores.	Concurrent	Spearman rank correlation	r_s_≥0.30^a)^	Confirmed
Student scores on earlier SCIPAIs will correlate with like-item final CPI scores.	Predictive	Spearman rank correlation	r_s_≥0.30^a)^	Confirmed

SCIPAI, Student and Clinical Instructor Performance Assessment Instrument; CEEs, clinical education experiences; CI, clinical instructor; CPI, Clinical Performance Instrument.

a) Cohen’s guidelines for social science research: medium effect r_s_ ≥0.30, large effect r_s_ ≥0.50.

## Footnotes

**Data availability**
Data files are available from https://doi.org/10.7910/DVN/DWRN8Q Dataset 1. Student and Clinical Instructor Performance Instrument validation study raw data.

## Supplementary materials

Supplement 1. PDF version of online Student and Clinical Instructor Performance Instrument.

Supplement 2. PDF version of online Clinical Performance Instrument.

Supplement 3. SCIPAI internal structure analyses.

Supplement 4. Audio recording of the abstract.
