# Supplementary material for: Validity of the formative physical therapy Student and Clinical Instructor Performance Assessment Instrument in the United States: a quasi-experimental, time-series study
Source: J Educ Eval Health Prof. 2025 Sep 26;22:26. doi: 10.3352/jeehp.2025.22.26 (PMC12688320; doi:10.3352/jeehp.2025.22.26)
Supplement: Supplementary file 2 — Supplement 1. PDF version of online Student and Clinical Instructor Performance Instrument. [file jeehp-22-26-suppl1.pdf]

Please check this box if you would like the DCE to contact you ☐

Student & Clinical Instructor Performance Assessment Tool (SCIPAI)

Student: ______________________________ CI : ___________________________

Clinical Site______________________________________

Clinical Week # _____ Date of meeting: ________________

<u>Student and CI complete ratings and comments; then compare with each other</u>

<u>Clinical Instructor Performance</u>:

Supervision __________ % *(Rate % of the time, 0-100%, CI manages effectively without needing cues)*
CI responsive to informal meetings, responsive to student needs/questions; CI provides appropriate balance of close/distant supervision; CI readily accessible

____

____

Feedback __________ % *(Rate % of the time, 0-100%, CI manages effectively without needing cues)*
CI answers questions regarding patient treatment or refers Intern to appropriate person or resources; CI provides positive reinforcement and critical comments – comments help with professional/personal growth

____

____

Kudos - CI performance from this past week

____

____

Goals - CI performance for next week

____

____

<u>Student Performance</u>:

Evaluation ________ % *(Rate % of the time, 0-100%, student manages effectively without needing cues)*
Student uses technically correct mechanics – i.e., MMT, ROM, sensory testing, functional mobility, etc. - in obtaining and documenting the data necessary to make treatment decisions

____

____

____

Treatment ______ % *(Rate % of the time, 0-100%, student manages effectively without needing cues)*
Student able to plan and complete appropriate treatments; provide creative treatment alternatives; justify treatment strategies – logic and evidence-based practice; competently apply treatment technique

____

____

____

Intellectual Property of the University of Dayton Doctor of Physical Therapy Program
Use only with express written permission.

Communication _______ % *(Rate % of the time, 0-100%, student manages effectively without needing cues)*
Student appropriately initiates and receives feedback from CI, peers (all other healthcare providers, including vendors), and patients/caregivers; appropriately interacts with CI, peers, patients/caregivers

____
____
____

Professional Behavior______ % *(Rate % of the time, 0-100%, student manages effectively without needing cues)*
Student demonstrates professional behavior in all situations: teamwork, initiative, punctuality, reliability, appropriate attire; integrity; compassion; caring; empathy; productive relationships; contributions to a positive work environment; accepts feedback, conflict management; values dignity of all; seeks and provides feedback

____
____
____

Kudos - Student performance from this past week

____
____
____

Goals - Student performance for next week

____
____
____

**Student Independence Performance Rating**	**Independence** (Rate % of caseload, 0-100%, student manages independently)
**Simple cases**	
**Complex cases**	
**Overall Caseload**	

To assist with ongoing dialogue between the University of Dayton, its students, and its clinical partner sites, students will input electronically on the SCIPAI App any SCIPAI data completed on paper. This will allow for DCE and CCCE review of the SCIPAI content. Access the SCIPAI App at:

Post SCIPAI Review Comments (optional)

____
____
____

Intellectual Property of the University of Dayton Doctor of Physical Therapy Program
Use only with express written permission.
