# Supplementary material for: Validity of the formative physical therapy Student and Clinical Instructor Performance Assessment Instrument in the United States: a quasi-experimental, time-series study
Source: J Educ Eval Health Prof. 2025 Sep 26;22:26. doi: 10.3352/jeehp.2025.22.26 (PMC12688320; doi:10.3352/jeehp.2025.22.26)
Supplement: Supplementary file 3 — Supplement 2. PDF version of online Clinical Performance Instrument. [file jeehp-22-26-suppl2.pdf]

PHYSICAL THERAPIST

CLINICAL PERFORMANCE INSTRUMENT

FOR STUDENTS

June 2006

American Physical Therapy Association
Department of Physical Therapy Education
1111 North Fairfax Street
Alexandria, Virginia 22314

APTA
American Physical Therapy Association

ISBN # 1-931369-25-9

© 1997, 2006 American Physical Therapy Association. All rights reserved.

For more information about this publication and other APTA publications, contact the American Physical Therapy Association, 1111 North Fairfax Street, Alexandria, VA 22314-1488.
[Publication No. E-42]

TABLE OF CONTENTS

Table of Contents 3
Copyright, Disclaimer, and Validity and Reliability in Using the Instrument 4
Instructions for the Use of the PT Clinical Performance Instrument 5
Introduction 5
Instructions for the Clinical Instructor 6
Instructions for the Student 7
Instructions for the ACCE/DCE 8
Components of the Form 10
Clinical Performance Instrument Information 14

Clinical Performance Criteria for the Physical Therapist Student 15

Professional Practice
1. Safety 15
2. Professional Behavior 16
3. Accountability*[1] 17
4. Communication* 18
5. Cultural Competence* 20
6. Professional Development 32

Patient Management
7. Clinical Reasoning* 19
8. Screening* 21
9. Examination* 22
10. Evaluation* 23
11. Diagnosis* and Prognosis* 24
12. Plan of Care* 25
13. Procedural Interventions 26
14. Educational Interventions* 27
15. Documentation* 28
16. Outcomes Assessment* 29
17. Financial Resources 30
18. Direction and Supervision of Personnel 31

Summative Comments 33
Evaluation Signatures (Midterm) 35
Evaluation Signatures (Final) 36
Glossary 37
Appendix A: Example: Completed Item for Final Experience (Competent) 46
Example: Completed Item for Final Experience (Not Competent) 47
Example: Completed Item for Intermediate Experience (Competent) 48
Appendix B: PT CPI Performance Criteria Matched with Evaluative Criteria for the Accreditation of Physical Therapist Programs 49
Appendix C: Definitions of Performance Dimensions and Rating Scale Anchors 50

[1] Terms used in this instrument are denoted by an asterisk (*) and can be found in the Glossary.

COPYRIGHT, DISCLAIMER, AND VALIDITY AND RELIABILITY IN USING THE INSTRUMENT

COPYRIGHT

The copyright in this Physical Therapist Clinical Performance Instrument (Instrument) is owned by the American Physical Therapy Association (APTA or Association).

Making a copy of the Instrument without the APTA's permission constitutes an infringement of copyright.

Preparing a work based on the Instrument by transforming, adapting, abridging, condensing, or otherwise adapting it without the APTA's permission constitutes an infringement of copyright.

Any person who infringes the APTA's copyright in the Instrument shall be subject to criminal liability in accordance with § 506 (Criminal offenses) of Title 17 and § 2319 (Criminal infringement of a copyright) of Title 18 of the United States Code.

DISCLAIMER

Parties use this Instrument at their own risk. The American Physical Therapy Association assumes no responsibility for any third party's use of this Instrument. The Association makes no representations concerning the suitability of this Instrument for any particular purpose, and it hereby explicitly disclaims any and all warranties concerning this Instrument when used by third parties.

VALIDITY AND RELIABILITY

The psychometric properties of the Instrument (ie, validity and reliability) are preserved <u>only</u> when it is used in accordance with the instructions that accompany it and <u>only</u> if the Instrument is not altered (by addition, deletion, revision, or otherwise) in any way.

CLINICAL PERFORMANCE INSTRUMENT

INTRODUCTION

- This instrument should only be used after completing the APTA web-based training for the Physical Therapist Clinical Performance Instrument (PT CPI) at www.apta/education **(TBD).**

- The PT CPI is applicable to a broad range of clinical settings and can be used throughout the continuum of clinical learning experiences.

- Every performance criterion* in this instrument is important to the overall assessment of clinical competence, and all criteria are observable in every clinical experience.

- All performance criteria should be rated based on observation of student performance relative to entry-level.

- The PT CPI from any previous student experience should not be shared with any subsequent experiences.

- The PT CPI consists of 18 performance criteria.

- Each performance criterion includes a list of sample behaviors, a section for midterm and final comments for each performance dimension, a rating scale consisting of a line with 6 defined anchors, and a significant concerns box for midterm and final evaluations.

- Terms used in this instrument are denoted by an asterisk (*) and can be found in the Glossary.

- Summative midterm and final comments and recommendations are provided at the end of the CPI.

- **Altering this instrument is a violation of copyright law**.

Instructions for the Clinical Instructor

- Sources of information to complete the PT CPI may include, but are not limited to, clinical instructors (CIs), other physical therapists, physical therapist assistants*, other professionals, patients/clients*, and students. Methods of data collection may include direct observation, videotapes, documentation review, role playing, interviews, standardized practical activities, portfolios, journals, computer-generated tests, and patient and outcome surveys.
- Prior to beginning to use the instrument in your clinical setting it would be useful to discuss and reach agreement on how the sample behaviors would be specifically demonstrated at entry-level by students in your clinical setting.
- The CI(s) will assess a student's performance and complete the instrument at midterm and final evaluation periods.
- The CI(s) reviews the completed instrument formally with the student at a minimum at the midterm evaluation and at the end of the clinical experience and signs the signature pages (midterm 35 and final 36) following each evaluation.
- Each academic institution is responsible for determining minimum performance expectations for successful completion of each clinical experience. Since CIs are not responsible for assigning grades it is essential for them to rate student performance based only on their direct observations of student performance.

Rating Scale

- The rating scale was designed to reflect a continuum of performance ranging from "Beginning Performance" to "Beyond Entry-Level Performance." Student performance should be described in relation to one or more of the six anchors. For example, consider the following rating on a selected performance criterion.

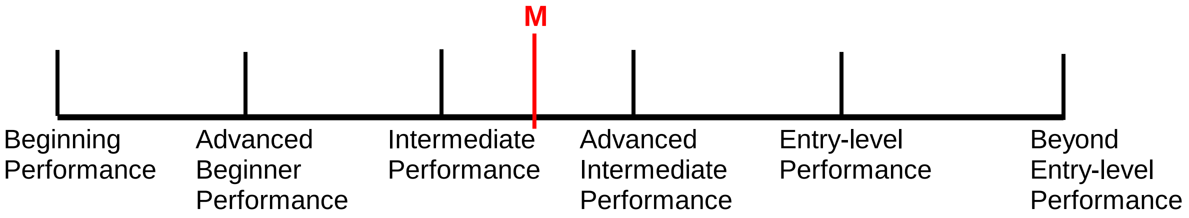

- The rating scale was not designed to be a visual analog scale. The vertical mark indicates that the student has exceeded the anchor definition of "intermediate performance," however the student has yet to satisfy the definition associated with "advanced intermediate performance." In order to place the rating on an anchor, all of the conditions of that level of the rating must be satisfied as provided in the description for each of the 6 anchors.

Instructions for the Student

- The student is expected to perform self-assessment based on CI feedback, student peer assessments, and patient/client assessments.
- The student self-assesses his/her performance on a separate copy of the instrument.
- The student reviews the completed instrument with the CI at the midterm evaluation and at the end of the clinical experience and signs the signature page (midterm 35 and final 36) following each evaluation.
- Each academic institution is responsible for determining minimum performance expectations for successful completion of each clinical experience. Since CIs are not responsible for assigning grades it is essential for them to rate student performance based only on their direct observations of student performance.

Rating Scale

- The rating scale was designed to reflect a continuum of performance ranging from “Beginning Performance” to “Beyond Entry-Level Performance.” Student performance should be described in relation to one or more of the six anchors. For example, consider the following rating on a selected performance criterion.

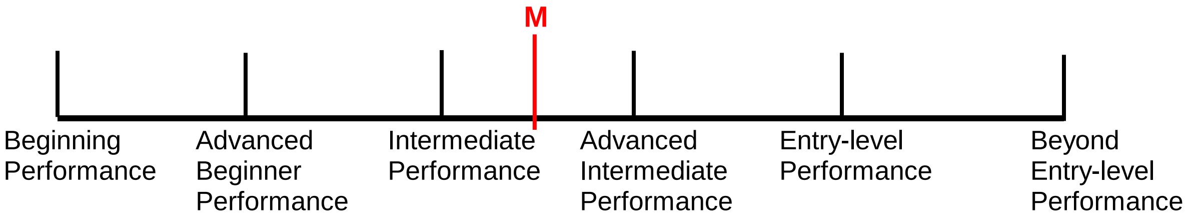

- The rating scale was not designed to be a visual analog scale. The vertical mark indicates that the student has exceeded the anchor definition of “intermediate performance” however the student has yet to satisfy the definition associated with “advanced intermediate performance.” In order to place the rating on an anchor, all of the conditions of that level of the rating must be satisfied as provided in the description for each of the 6 anchors.

Instructions for the Academic Coordinator/Director of Clinical Education (ACCE/DCE*)

- A physical therapist (PT) student assessment* system evaluates knowledge, skills, and attitudes and incorporates multiple sources of information to make decisions about readiness to practice.
- Sources of information may include clinical performance evaluations of students, classroom performance evaluations, students' self-assessments, peer assessments, and patient assessments. The system is intended to enable clinical educators and academic faculty to obtain a comprehensive perspective of students' progress through the curriculum and competence* to practice at entry-level. The uniform adoption and consistent use of this instrument will ensure that all practitioners entering practice have demonstrated a core set of clinical attributes.
- The ACCE/DCE* reviews the completed form at the end of the clinical experience and assigns a grade or pass/fail according to institution policy.

Rating Scale

- The rating scale was designed to reflect a continuum of performance ranging from "Beginning Performance" to "Beyond Entry-Level Performance." Student performance should be described in relation to one or more of the six anchors. For example, consider the following rating on a selected performance criterion.

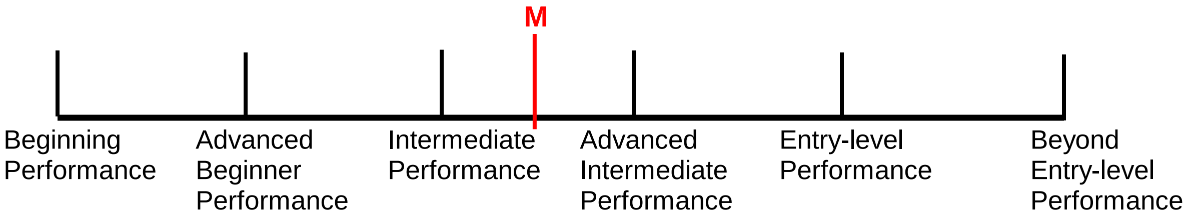

- The rating scale was not designed to be a visual analog scale. The vertical mark indicates that the student has exceeded the anchor definition of "intermediate performance," however the student has yet to satisfy the definition associated with "advanced intermediate performance." In order to place the rating on an anchor, all of the conditions of that level of the rating must be satisfied as provided in the description for each of the 6 anchors.
- Attempts to quantify a rating on the scale in millimeters or as a percentage would be considered an invalid use of the assessment tool. For example, a given academic institution may require their students to achieve a minimum student rating of "intermediate performance" by the conclusion of an initial clinical experience. It was not the intention of the developers to establish uniform grading criteria given the unique curricular design of each academic institution.
- Each academic institution is responsible for determining minimum performance expectations for successful completion of each clinical experience. Since clinical instructors (CIs) are not responsible for assigning grades it is essential for them to rate student performance based only on their direct observations of student performance. It would be inappropriate for the ACCE/DCE to provide a pre-marked PT CPI with minimum performance expectations, send an additional page of information that identify specific marked expectations, or add/delete items from PT CPI.

Determining a Grade

- Each academic institution determines what constitutes satisfactory performance. The guide below is provided to assist the program in identifying what is expected for the student's performance depending upon their level of education* and clinical experience within the program.

- ➢ First clinical experience: Depending upon your academic curriculum, ratings of student performance may be expected in the first two intervals between beginning clinical performance,* advanced beginner performance, and intermediate clinical performance.
- ➢ Intermediate clinical experiences: Depending upon your academic curriculum, student performance ratings are expected to progress along the continuum ranging from a minimum of advanced beginner clinical performance (interval 2) to advanced intermediate clinical performance* (interval 4). The ratings on the performance criteria will be dependent upon the clinical setting, level of didactic and clinical experience within the curriculum, and expectations of the clinical site and the academic program.
- ➢ Final clinical experience*:* Students should achieve ratings of entry-level or beyond (interval 5) for all 18 performance criteria.

- At the conclusion of a clinical experience, grading decisions made by the ACCE/DCE, may also consider:
  - clinical setting,
  - experience with patients or clients* in that setting,
  - relative weighting or importance of each performance criterion,
  - expectations for the clinical experience,
  - progression of performance from midterm to final evaluations,
  - level of experience within the didactic and clinical components,
  - whether or not “significant concerns” box was checked, and
  - the congruence between the CI’s narrative midterm and final comments related to the five performance dimensions and the ratings provided.

COMPONENTS OF THE FORM

Performance Criteria*

- The 18 performance criteria* describe the essential aspects of professional practice of a physical therapist* clinician performing at entry-level.
- The performance criteria are grouped by the aspects of practice that they represent.
- Items 1-6 are related to professional practice, items 7-15 address patient management, and items 16-18 address practice management*.

Red Flag Item

- A flag ( ) to the left of a performance criterion indicates a "red-flag" item.
- The five "red-flag" items (numbered 1, 2, 3, 4, and 7) are considered foundational elements in clinical practice.
- Students may progress more rapidly in the "red flag" areas than other performance criteria.
- Significant concerns related to a performance criterion that is a red-flag item warrants immediate attention, more expansive documentation*, and a telephone call to the ACCE/DCE*. Possible outcomes from difficulty in performance with a red-flag item may include remediation, extension of the experience with a learning contract, and/or dismissal from the clinical experience.

Sample Behaviors

- The sample of commonly observed behaviors (denoted with lower-case letters in shaded boxes) for each criterion are used to guide assessment* of students' competence relative to the performance criteria.
- Given the diversity and complexity of clinical practice, it must be emphasized that ***the sample behaviors provided are not meant to be an exhaustive list.***
- There may be additional or alternative behaviors relevant and critical to a given clinical setting and all listed behaviors need not be present to rate student performance at the various levels.
- Sample behaviors are not listed in order of priority, but most behaviors are presented in logical order.

Midterm and Final Comments

- The clinical instructor* must provide descriptive narrative comments for all performance criteria.
- For each performance criterion, space is provided for written comments for midterm and final ratings.
- Each of the five performance dimensions (supervision/guidance*, quality*, complexity*, consistency*, and efficiency*) are common to all types and levels of performance and should be addressed in providing written comments.

Performance Dimensions

- **Supervision/guidance*** refers to the level and extent of assistance required by the student to achieve entry-level performance.
  - As a student progresses through clinical education experiences*, the degree of supervision/guidance needed is expected to progress from 100% supervision to being capable of independent performance with consultation* and may vary with the complexity of the patient or environment.

- **Quality*** refers to the degree of knowledge and skill proficiency demonstrated.
  - As a student progresses through clinical education experiences, quality should range from demonstration of limited skill to a skilled or highly skilled performance.

- **Complexity*** refers to the number of elements that must be considered relative to the patient*, task, and/or environment.
  - As a student progresses through clinical education experiences, the level of complexity of tasks, patient management, and the environment should increase, with fewer elements being controlled by the CI.

- **Consistency*** refers to the frequency of occurrences of desired behaviors related to the performance criterion.
  - As a student progresses through clinical education experiences, consistency of quality performance is expected to progress from infrequently to routinely.

- **Efficiency*** refers to the ability to perform in a cost-effective and timely manner.
  - As the student progresses through clinical education experiences, efficiency should progress from a high expenditure of time and effort to economical and timely performance.

Rating Student Performance

- Each performance criterion is rated relative to entry-level practice as a physical therapist.
- The rating scale consists of a horizontal line with 6 vertical lines defining anchors at each end and at four intermediate points along that line.
- The 6 vertical lines define the borders of five intervals.
- Rating marks may be placed on the 6 vertical lines or anywhere within the five intervals.
- The same rating scale is used for midterm evaluations and final evaluations.
- Place one vertical line on the rating scale at the appropriate point indicating the midterm evaluation rating and label it with an "**M**".
- Place one vertical line on the rating scale at the appropriate point indicating the final evaluation rating and label it with an "**F**".
- Placing a rating mark on a vertical line indicates the student's performance matches the definition attached to that particular vertical line.
- Placing a rating mark in an interval indicates that the student's performance is somewhere between the definitions attached to the vertical marks defining that interval.
- For completed examples of how to mark the rating scale, refer to *Appendix A: Examples)*.

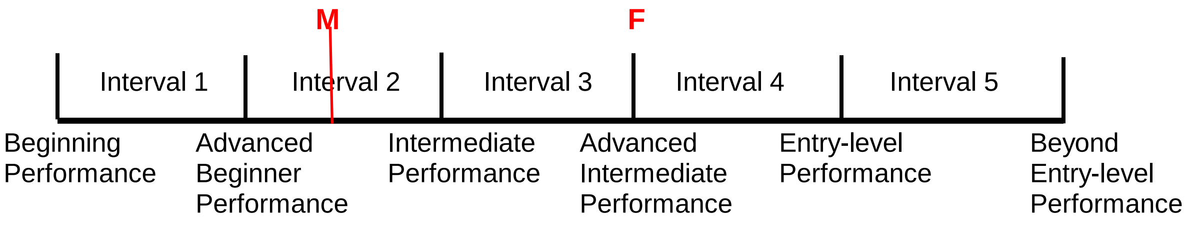

Anchor Definitions

Beginning performance*:

- A student who requires close clinical supervision 100% of the time managing patients with constant monitoring and feedback, even with patients with simple conditions.
- At this level, performance is inconsistent and clinical reasoning* is performed in an inefficient manner.
- Performance reflects little or no experience.
- The student does not carry a caseload.

Advanced beginner performance*:

- A student who requires clinical supervision 75% – 90% of the time managing patients with simple conditions, and 100% of the time managing patients with complex conditions.
- At this level, the student demonstrates consistency in developing proficiency with simple tasks (eg, medical record review, goniometry, muscle testing, and simple interventions), but is unable to perform skilled examinations, interventions, and clinical reasoning skills.
- The student may begin to share a caseload with the clinical instructor.

Intermediate performance*:

- A student who requires clinical supervision less than 50% of the time managing patients with simple conditions, and 75% of the time managing patients with complex conditions.
- At this level, the student is proficient with simple tasks and is developing the ability to consistently perform skilled examinations, interventions, and clinical reasoning.
- The student is **capable of** maintaining 50% of a full-time physical therapist's caseload.

Advanced intermediate performance*:

- A student who requires clinical supervision less than 25% of the time managing new patients or patients with complex conditions and is independent managing patients with simple conditions.
- At this level, the student is consistent and proficient in simple tasks and requires only occasional cueing for skilled examinations, interventions, and clinical reasoning.
- The student is **capable of** maintaining 75% of a full-time physical therapist's caseload.

Entry-level performance*:

- A student who is **capable of** functioning without guidance or clinical supervision managing patients with simple or complex conditions.
- At this level, the student is consistently proficient and skilled in simple and complex tasks for skilled examinations, interventions, and clinical reasoning.
- Consults with others and resolves unfamiliar or ambiguous situations.
- The student is **capable of** maintaining 100% of a full-time physical therapist's caseload in a cost effective manner.

Beyond entry-level performance*:

- A student who is **capable of** functioning without clinical supervision or guidance in managing patients with simple or highly complex conditions, and is able to function in unfamiliar or ambiguous situations.
- At this level, the student is consistently proficient at highly skilled examinations, interventions, and clinical reasoning, and is a capable of serving as a consultant or resource for others.
- The student is **capable of** maintaining 100% of a full-time physical therapist's caseload and seeks to assist others where needed.
- The student is capable of supervising others.
- The student willingly assumes a leadership role* for managing patients with more difficult or complex conditions.

- Actively contributes to the enhancement of the clinical facility with an expansive view of physical therapy practice and the profession.

Significant Concerns Box

- Checking this box (☐) indicates that the student's performance on this criterion is unacceptable for this clinical experience.
- When the Significant Concerns Box is checked, written comments to substantiate the concern, additional documentation such as a critical incident form and learning contract are required with a phone call (☎) placed to the ACCE.
- The significant concerns box provides an early warning system to identify student performance problems thereby enabling the CI, student, and ACCE/DCE to determine a mechanism for remediation, if appropriate.
- A box is provided for midterm and final assessments*.

Summative Comments

- Summative comments should be used to provide a global perspective of the student's performance across all 18 criteria at midterm and final evaluations.
- The summative comments, located after the last performance criterion, provide a section for the rater to comment on the overall strengths, areas requiring further development, other general comments, and any specific recommendations with respect to the learner's needs, interests, planning, or performance.
- Comments should be based on the student's performance relative to stated objectives* for the clinical experience.

CLINICAL PERFORMANCE INSTRUMENT INFORMATION

STUDENT INFORMATION (Student to Complete)

Student's Name:____

Date of Clinical Experience:________________________Course Number:__________________________

E-mail:____

Total Number of Days Absent: ___________________________________

Specify Clinical Experience(s)/Rotation(s) Completed:

_______Acute Care/Inpatient	_______Private Practice
_______Ambulatory Care/Outpatient	_______Rehab/Sub-Acute Rehab
_______ECF/Nursing Home/SNF	_______School/Pre-school
_______Federal/State/County Health	_______Wellness/Prevention/Fitness
_______Industrial/Occupational Health	_______Other; specify _______________

ACADEMIC PROGRAM INFORMATION (Program to Complete)

Name of Academic Institution: ____

Address:____
(Department) (Street)

____
(City) (State/Province) (Zip)

Phone: ___________________________ext.__________Fax: ____________________________________

E-mail: ____Website:_________________________________

CLINICAL EDUCATION SITE INFORMATION (Clinical Site to Complete)

Name of Clinical Site: ____

Address: ____
(Department) (Street)

____
(City) (State/Province) (Zip)

Phone: ___________________________ext.__________Fax: ____________________________________

E-mail: ____Website:_________________________________

Clinical Instructor's* Name: ____

Clinical Instructor's Name: ____

Clinical Instructor's Name: ____

Center Coordinator of Clinical Education's Name: ____

PROFESSIONAL PRACTICE

SAFETY

1. Practices in a safe manner that minimizes the risk to patient, self, and others.

SAMPLE BEHAVIORS

a. Establishes and maintains safe working environment.
b. Recognizes physiological and psychological changes in patients* and adjusts patient interventions* accordingly.
c. Demonstrates awareness of contraindications and precautions of patient intervention.
d. Ensures the safety of self, patient, and others throughout the clinical interaction (eg, universal precautions, responding and reporting emergency situations, etc).
e. Requests assistance when necessary.
f. Uses acceptable techniques for safe handling of patients (eg, body mechanics, guarding, level of assistance, etc.).
g. Demonstrates knowledge of facility safety policies and procedures.

MIDTERM COMMENTS: (Provide comments based on the performance dimensions including supervision/guidance*, quality*, complexity*, consistency*, and efficiency*.)

FINAL COMMENTS: (Provide comments based on the performance dimensions including supervision/guidance*, quality*, complexity*, consistency*, and efficiency*.)

Rate this student's clinical performance based on the sample behaviors and comments above:

Beginning Performance*	Advanced Beginner Performance*	Intermediate Performance*	Advanced Intermediate Performance*	Entry-level Performance*	Beyond Entry-level Performance*

Significant Concerns: **If performance on this criterion is unacceptable, check the box and call the ACCE/DCE.**

☎ **Midterm** ☐ ☎ **Final** ☐

PROFESSIONAL BEHAVIOR

2. Demonstrates professional behavior in all situations.

SAMPLE BEHAVIORS

a. Demonstrates initiative (eg, arrives well prepared, offers assistance, seeks learning opportunities).
b. Is punctual and dependable.
c. Wears attire consistent with expectations of the practice setting.
d. Demonstrates integrity* in all interactions.
e. Exhibits caring*, compassion*, and empathy* in providing services to patients.
f. Maintains productive working relationships with patients, families, CI, and others.
g. Demonstrates behaviors that contribute to a positive work environment.
h. Accepts feedback without defensiveness.
i. Manages conflict in constructive ways.
j. Maintains patient privacy and modesty.
k. Values the dignity of patients as individuals.
l. Seeks feedback from clinical instructor related to clinical performance.
m. Provides effective feedback to CI related to clinical/teaching mentoring.

MIDTERM COMMENTS: (Provide comments based on the performance dimensions including supervision/guidance*, quality*, complexity*, consistency*, and efficiency*.)

FINAL COMMENTS: (Provide comments based on the performance dimensions including supervision/guidance*, quality*, complexity*, consistency*, and efficiency.)

Rate this student's clinical performance based on the sample behaviors and comments above:

Beginning Performance	Advanced Beginner Performance	Intermediate Performance	Advanced Intermediate Performance	Entry-level Performance	Beyond Entry-level Performance

Significant Concerns: If performance on this criterion is unacceptable, check the box and call the ACCE/DCE.

☎ **Midterm** ☐ ☎ **Final** ☐

PROFESSIONAL PRACTICE

ACCOUNTABILITY*

3. Practices in a manner consistent with established legal and professional standards and ethical guidelines.

SAMPLE BEHAVIORS

a. Places patient's needs above self interests.
b. Identifies, acknowledges, and accepts responsibility for actions and reports errors.
c. Takes steps to remedy errors in a timely manner.
d. Abides by policies and procedures of the practice setting (eg, OSHA, HIPAA, PIPEDA [Canada], etc.)
e. Maintains patient confidentiality.
f. Adheres to legal practice standards including all federal, state/province, and institutional regulations related to patient care and fiscal management.*
g. Identifies ethical or legal concerns and initiates action to address the concerns.
h. Displays generosity as evidenced in the use of time and effort to meet patient needs.
i. Recognize the need for physical therapy services to underserved and under represented populations.
j. Strive to provide patient/client services that go beyond expected standards of practice.

MIDTERM COMMENTS: (Provide comments based on the performance dimensions including supervision/guidance*, quality*, complexity*, consistency*, and efficiency*.)

FINAL COMMENTS: (Provide comments based on the performance dimensions including supervision/guidance*, quality*, complexity*, consistency*, and efficiency*.)

Rate this student's clinical performance based on the sample behaviors and comments above:

Beginning Performance	Advanced Beginner Performance	Intermediate Performance	Advanced Intermediate Performance	Entry-level Performance	Beyond Entry-level Performance

Significant Concerns: **If performance on this criterion is unacceptable, check the box and call the ACCE/DCE.**

☎ **Midterm** ☐ ☎ **Final** ☐

PROFESSIONAL PRACTICE

COMMUNICATION*

4. Communicates in ways that are congruent with situational needs.

SAMPLE BEHAVIORS

a. Communicates, verbally and nonverbally, in a professional and timely manner.
b. Initiates communication* in difficult situations.
c. Selects the most appropriate person(s) with whom to communicate.
d. Communicates respect for the roles* and contributions of all participants in patient care.
e. Listens actively and attentively to understand what is being communicated by others.
f. Demonstrates professionally and technically correct written and verbal communication without jargon.
g. Communicates using nonverbal messages that are consistent with intended message.
h. Engages in ongoing dialogue with professional peers or team members.
i. Interprets and responds to the nonverbal communication of others.
j. Evaluates effectiveness of his/her communication and modifies communication accordingly.
k. Seeks and responds to feedback from multiple sources in providing patient care.
l. Adjust style of communication based on target audience.
m. Communicates with the patient using language the patient can understand (eg, translator, sign language, level of education*, cognitive* impairment*, etc).

MIDTERM COMMENTS: (Provide comments based on the performance dimensions including supervision/guidance*, quality*, complexity*, consistency*, and efficiency*.)

FINAL COMMENTS: (Provide comments based on the performance dimensions including supervision/guidance*, quality*, complexity*, consistency*, and efficiency*.)

Rate this student's clinical performance based on the sample behaviors and comments above:

Beginning Performance	Advanced Beginner Performance	Intermediate Performance	Advanced Intermediate Performance	Entry-level Performance	Beyond Entry-level Performance

Significant Concerns: If performance on this criterion is unacceptable, check the box and call the ACCE/DCE.

☎ **Midterm** ☐ ☎ **Final** ☐

PROFESSIONAL PRACTICE

CULTURAL COMPETENCE*

5. Adapts delivery of physical therapy services with consideration for patients' differences, values, preferences, and needs.

SAMPLE BEHAVIORS

a. Incorporates an understanding of the implications of individual and cultural differences and adapts behavior accordingly in all aspects of physical therapy services.
b. Communicates with sensitivity by considering differences in race/ethnicity, religion, gender, age, national origin, sexual orientation, and disability* or health status.*
c. Provides care in a nonjudgmental manner when the patients' beliefs and values conflict with the individual's belief system.
d. Discovers, respects, and highly regards individual differences, preferences, values, life issues, and emotional needs within and among cultures.
e. Values the socio-cultural, psychological, and economic influences on patients and clients* and responds accordingly.
f. Is aware of and suspends own social and cultural biases.

MIDTERM COMMENTS: (Provide comments based on the performance dimensions including supervision/guidance*, quality*, complexity*, consistency*, and efficiency*.)

FINAL COMMENTS: (Provide comments based on the performance dimensions including supervision/guidance*, quality*, complexity*, consistency*, and efficiency*.)

Rate this student's clinical performance based on the sample behaviors and comments above:

Beginning Performance	Advanced Beginner Performance	Intermediate Performance	Advanced Intermediate Performance	Entry-level Performance	Beyond Entry-level Performance

Significant Concerns: **If performance on this criterion is unacceptable, check the box and call the ACCE/DCE.**

☎ **Midterm** ☐ ☎ **Final** ☐

PROFESSIONAL PRACTICE

PROFESSIONAL DEVELOPMENT

6. Participates in self-assessment to improve clinical and professional performance.

SAMPLE BEHAVIORS

a. Identifies strengths and limitations in clinical performance.
b. Seeks guidance as necessary to address limitations.
c. Uses self-evaluation, ongoing feedback from others, inquiry, and reflection to conduct regular ongoing self-assessment to improve clinical practice and professional development.
d. Acknowledges and accepts responsibility for and consequences of his or her actions.
e. Establishes realistic short and long-term goals in a plan for professional development.
f. Seeks out additional learning experiences to enhance clinical and professional performance.
g. Discusses progress of clinical and professional growth.
h. Accepts responsibility for continuous professional learning.
i. Discusses professional issues related to physical therapy practice.
j. Participates in professional activities beyond the practice environment.
k. Provides to and receives feedback from peers regarding performance, behaviors, and goals.
l. Provides current knowledge and theory (in-service, case presentation, journal club, projects, systematic data collection, etc) to achieve optimal patient care.

MIDTERM COMMENTS: (Provide comments based on the performance dimensions including supervision/guidance*, quality*, complexity*, consistency*, and efficiency*.)

FINAL COMMENTS: (Provide comments based on the performance dimensions including supervision/guidance*, quality*, complexity*, consistency*, and efficiency*.)

Rate this student's clinical performance based on the sample behaviors and comments above:

Beginning Performance	Advanced Beginner Performance	Intermediate Performance	Advanced Intermediate Performance	Entry-level Performance	Beyond Entry-level Performance

Significant Concerns: **If performance on this criterion is unacceptable, check the box and call the ACCE/DCE.**

☏ **Midterm** ☐ ☏ **Final** ☐

PATIENT MANAGEMENT

CLINICAL REASONING*

7. Applies current knowledge, theory, clinical judgment, and the patient's values and perspective in patient management.

SAMPLE BEHAVIORS

a. Presents a logical rationale (cogent and concise arguments) for clinical decisions.
b. Makes clinical decisions within the context of ethical practice.
c. Utilizes information from multiple data sources to make clinical decisions (eg, patient and caregivers*, health care professionals, hooked on evidence, databases, medical records).
d. Seeks disconfirming evidence in the process of making clinical decisions.
e. Recognizes when plan of care* and interventions are ineffective, identifies areas needing modification, and implements changes accordingly.
f. Critically evaluates published articles relevant to physical therapy and applies them to clinical practice.
g. Demonstrates an ability to make clinical decisions in ambiguous situations or where values may be in conflict.
h. Selects interventions based on the best available evidence, clinical expertise, and patient preferences.
i. Assesses patient response to interventions using credible measures.
j. Integrates patient needs and values in making decisions in developing the plan of care.
k. Clinical decisions focus on the whole person rather than the disease.
l. Recognizes limits (learner and profession) of current knowledge, theory, and judgment in patient management.

MIDTERM COMMENTS: (Provide comments based on the performance dimensions including supervision/guidance*, quality*, complexity*, consistency*, and efficiency*.)

FINAL COMMENTS: (Provide comments based on the performance dimensions including supervision/guidance*, quality*, complexity*, consistency*, and efficiency*.)

Rate this student's clinical performance based on the sample behaviors and comments above:

Beginning Performance	Advanced Beginner Performance	Intermediate Performance	Advanced Intermediate Performance	Entry-level Performance	Beyond Entry-level Performance

Significant Concerns: If performance on this criterion is unacceptable, check the box and call the ACCE/DCE.

☏ **Midterm** ☐ ☏ **Final** ☐

SCREENING*

8. Determines with each patient encounter the patient's need for further examination or consultation* by a physical therapist* or referral to another health care professional.

SAMPLE BEHAVIORS

a. Utilizes test and measures sensitive to indications for physical therapy intervention.
b. Advises practitioner about indications for intervention.
c. Reviews medical history* from patients and other sources (eg, medical records, family, other health care staff).
d. Performs a system review and recognizes clusters (historical information, signs and symptoms) that would preclude interventions due to contraindications or medical emergencies.
e. Selects the appropriate screening* tests and measurements.
f. Conducts tests and measurements appropriately.
g. Interprets tests and measurements accurately.
h. Analyzes and interprets the results and determines whether there is a need for further examination or referral to other services.
i. Chooses the appropriate service and refers the patient in a timely fashion, once referral or consultation is deemed necessary
j. Conducts musculoskeletal, neuromuscular, cardiopulmonary, and integumentary systems screening at community sites.

MIDTERM COMMENTS: (Provide comments based on the performance dimensions including supervision/guidance*, quality*, complexity*, consistency*, and efficiency*.)

FINAL COMMENTS: (Provide comments based on the performance dimensions including supervision/guidance*, quality*, complexity*, consistency*, and efficiency*.)

Rate this student's clinical performance based on the sample behaviors and comments above:

Beginning Performance	Advanced Beginner Performance	Intermediate Performance	Advanced Intermediate Performance	Entry-level Performance	Beyond Entry-level Performance

Significant Concerns: **If performance on this criterion is unacceptable, check the box and call the ACCE/DCE.**

☏ **Midterm** ☐ ☏ **Final** ☐

PATIENT MANAGEMENT

EXAMINATION*

9. Performs a physical therapy patient examination using evidenced-based* tests and measures.

SAMPLE BEHAVIORS

a. Obtains a history* from patients and other sources as part of the examination.*
b. Utilizes information from history and other data (eg, laboratory, diagnostic tests and pharmacological information) to formulate initial hypothesis and prioritize selection of test and measures.
c. Performs systems review.
d. Selects evidence-based tests and measures* that are relevant to the history, chief complaint, and screening.
   Tests and measures* (listed alphabetically) include, but are not limited to, the following: a) aerobic capacity, b) anthropometric characteristics, c) arousal, mentation, and cognition, d) assistive and adaptive devices*, e) community and work (job, school, or play) reintegration, f) cranial nerve integrity, g) environmental, home, and work barriers, h) ergonomics and body mechanics, i) gait, assisted locomotion, and balance, j) integumentary integrity, k) joint integrity and mobility, l) motor function*, m) muscle performance (including strength, power, and endurance), n) neuromotor development and sensory integration, o) orthotic, protective, and supportive devices, p) pain, q), posture, r) prosthetic requirements, s) range of motion, t) reflex integrity, u) self-care and home management (including activities of daily living and instrumental activities of daily living), v) sensory integration (including proprioception and kinesthesia), and w) ventilation, respiration, and circulation.
e. Conducts tests and measures accurately and proficiently.
f. Sequences tests and measures in a logical manner to optimize efficiency*.
g. Adjusts tests and measures according to patient's response.
h. Performs regular reexaminations* of patient status.
i. Performs an examination using evidence based test and measures.

MIDTERM COMMENTS: (Provide comments based on the performance dimensions including supervision/guidance*, quality*, complexity*, consistency*, and efficiency*.)

FINAL COMMENTS: (Provide comments based on the performance dimensions including supervision/guidance*, quality*, complexity*, consistency*, and efficiency*.)

Rate this student's clinical performance based on the sample behaviors and comments above:

Beginning Performance	Advanced Beginner Performance	Intermediate Performance	Advanced Intermediate Performance	Entry-level Performance	Beyond Entry-level Performance

Significant Concerns: **If performance on this criterion is unacceptable, check the box and call the ACCE/DCE.**

☎ **Midterm** ☐ ☎ **Final** ☐

PATIENT MANAGEMENT

EVALUATION*

10. Evaluates data from the patient examination (history, systems review, and tests and measures) to make clinical judgments.

SAMPLE BEHAVIORS

a. Synthesizes examination data and identifies pertinent impairments, functional limitations* and quality of life. [WHO – ICF Model for Canada]
b. Makes clinical judgments based on data from examination (history, system review, tests and measurements).
c. Reaches clinical decisions efficiently.
d. Cites the evidence to support a clinical decision.

MIDTERM COMMENTS: (Provide comments based on the performance dimensions including supervision/guidance*, quality*, complexity*, consistency*, and efficiency*.)

FINAL COMMENTS: (Provide comments based on the performance dimensions including supervision/guidance*, quality*, complexity*, consistency*, and efficiency*.)

Rate this student's clinical performance based on the sample behaviors and comments above:

Beginning Performance	Advanced Beginner Performance	Intermediate Performance	Advanced Intermediate Performance	Entry-level Performance	Beyond Entry-level Performance

Significant Concerns: If performance on this criterion is unacceptable, check the box and call the ACCE/DCE.

☎ **Midterm** ☐ ☎ **Final** ☐

PATIENT MANAGEMENT

DIAGNOSIS* AND PROGNOSIS*

11. Determines a diagnosis* and prognosis* that guides future patient management.

SAMPLE BEHAVIORS

a. Establishes a diagnosis for physical therapy intervention and list for differential diagnosis*.
b. Determines a diagnosis that is congruent with pathology, impairment, functional limitation, and disability.
c. Integrates data and arrives at an accurate prognosis* with regard to intensity and duration of interventions and discharge* status.
d. Estimates the contribution of factors (eg, preexisting health status, co-morbidities, race, ethnicity, gender, age, health behaviors) on the effectiveness of interventions.
e. Utilizes the research and literature to identify prognostic indicators (co-morbidities, race, ethnicity, gender, health behaviors, etc) that help predict patient outcomes.

MIDTERM COMMENTS: (Provide comments based on the performance dimensions including supervision/guidance*, quality*, complexity*, consistency*, and efficiency*.)

FINAL COMMENTS: (Provide comments based on the performance dimensions including supervision/guidance*, quality*, complexity*, consistency*, and efficiency*.)

Rate this student's clinical performance based on the sample behaviors and comments above:

Beginning Performance	Advanced Beginner Performance	Intermediate Performance	Advanced Intermediate Performance	Entry-level Performance	Beyond Entry-level Performance

Significant Concerns: If performance on this criterion is unacceptable, check the box and call the ACCE/DCE.

☏ **Midterm** ☐ ☏ **Final** ☐

PATIENT MANAGEMENT

PLAN OF CARE*

12. Establishes a physical therapy plan of care* that is safe, effective, patient-centered, and evidence-based.

SAMPLE BEHAVIORS

a. Establishes goals* and desired functional outcomes* that specify expected time durations.
b. Establishes a physical therapy plan of care* in collaboration with the patient, family, caregiver, and others involved in the delivery of health care services.
c. Establishes a plan of care consistent with the examination and evaluation.*
d. Selects interventions based on the best available evidence and patient preferences.
e. Follows established guidelines (eg, best practice, clinical pathways, and protocol) when designing the plan of care.
f. Progresses and modifies plan of care and discharge planning based on patient responses.
g. Identifies the resources needed to achieve the goals included in the patient care.
h. Implements, monitors, adjusts, and periodically re-evaluate a plan of care and discharge planning.
i. Discusses the risks and benefits of the use of alternative interventions with the patient.
j. Identifies patients who would benefit from further follow-up.
k. Advocates for the patients' access to services.

MIDTERM COMMENTS: (Provide comments based on the performance dimensions including supervision/guidance*, quality*, complexity*, consistency*, and efficiency*.)

FINAL COMMENTS: (Provide comments based on the performance dimensions including supervision/guidance*, quality*, complexity*, consistency*, and efficiency*.)

Rate this student's clinical performance based on the sample behaviors and comments above:

Beginning Performance	Advanced Beginner Performance	Intermediate Performance	Advanced Intermediate Performance	Entry-level Performance	Beyond Entry-level Performance

Significant Concerns: **If performance on this criterion is unacceptable, check the box and call the ACCE/DCE.**

☏ **Midterm** ☐ ☏ **Final** ☐

PROCEDURAL INTERVENTIONS*

13. Performs physical therapy interventions* in a competent manner.

SAMPLE BEHAVIORS

a. Performs interventions* safely, effectively, efficiently, fluidly, and in a coordinated and technically competent* manner.
   Interventions (listed alphabetically) include, but not limited to, the following: a) airway clearance techniques, b) debridement and wound care, c) electrotherapeutic modalities, d) functional training in community and work (job, school, or play) reintegration (including instrumental activities of daily living, work hardening, and work conditioning), e) functional training in self-care and home management (including activities of daily living and instrumental activities of daily living), f) manual therapy techniques*: spinal/peripheral joints (thrust/non-thrust), g) patient-related instruction, h) physical agents and mechanical modalities, i) prescription, application, and as appropriate fabrication of adaptive, assistive, orthotic, protective, and supportive devices and equipment, and j) therapeutic exercise (including aerobic conditioning).
b. Performs interventions consistent with the plan of care.
c. Utilizes alternative strategies to accomplish functional goals.
d. Follows established guidelines when implementing an existing plan of care.
e. Provides rationale for interventions selected for patients presenting with various diagnoses.
f. Adjusts intervention strategies according to variables related to age, gender, co-morbidities, pharmacological interventions, etc.
g. Assesses patient response to interventions and adjusts accordingly.
h. Discusses strategies for caregivers to minimize risk of injury and to enhance function.
i. Considers prevention*, health, wellness* and fitness* in developing a plan of care for patients with musculoskeletal, neuromuscular, cardiopulmonary, and integumentary system problems.
j. Incorporates the concept of self-efficacy in wellness and health promotion.*

MIDTERM COMMENTS: (Provide comments based on the performance dimensions including supervision/guidance*, quality*, complexity*, consistency*, and efficiency*.)

FINAL COMMENTS: (Provide comments based on the performance dimensions including supervision/guidance*, quality*, complexity*, consistency*, and efficiency*.)

Rate this student's clinical performance based on the sample behaviors and comments above:

Beginning Performance	Advanced Beginner Performance	Intermediate Performance	Advanced Intermediate Performance	Entry-level Performance	Beyond Entry-level Performance

Significant Concerns: If performance on this criterion is unacceptable, check the box and call the ACCE/DCE.

☏ **Midterm** ☐ ☏ **Final** ☐

EDUCATIONAL INTERVENTIONS*

14. Educates* others (patients, caregivers, staff, students, other health care providers*, business and industry representatives, school systems) using relevant and effective teaching methods.

SAMPLE BEHAVIORS

a. Identifies and establishes priorities for educational needs in collaboration with the learner.
b. Identifies patient learning style (eg, demonstration, verbal, written).
c. Identifies barriers to learning (eg, literacy, language, cognition).
d. Modifies interaction based on patient learning style.
e. Instructs patient, family members and other caregivers regarding the patient's condition, intervention and transition to his or her role at home, work, school or community.
f. Ensures understanding and effectiveness of recommended ongoing program.
g. Tailors interventions with consideration for patient family situation and resources.
h. Provides patients with the necessary tools and education* to manage their problem.
i. Determines need for consultative services.
j. Applies physical therapy knowledge and skills to identify problems and recommend solutions in relevant settings (eg, ergonomic evaluations, school system assessments*, corporate environmental assessments*).
k. Provides education and promotion of health, wellness, and fitness.

MIDTERM COMMENTS: (Provide comments based on the performance dimensions including supervision/guidance*, quality*, complexity*, consistency*, and efficiency*.)

FINAL COMMENTS: (Provide comments based on the performance dimensions including supervision/guidance*, quality*, complexity*, consistency*, and efficiency*.)

Rate this student's clinical performance based on the sample behaviors and comments above:

Beginning Performance	Advanced Beginner Performance	Intermediate Performance	Advanced Intermediate Performance	Entry-level Performance	Beyond Entry-level Performance

Significant Concerns: If performance on this criterion is unacceptable, check the box and call the ACCE/DCE.

☏ **Midterm** ☐ ☏ **Final** ☐

PATIENT MANAGEMENT

DOCUMENTATION*

15. Produces quality documentation* in a timely manner to support the delivery of physical therapy services.

SAMPLE BEHAVIORS

a. Selects relevant information to document the delivery of physical therapy care.
b. Documents all aspects of physical therapy care, including screening, examination, evaluation, plan of care, intervention, response to intervention, discharge planning, family conferences, and communication* with others involved in the delivery of care.
c. Produces documentation (eg, electronic, dictation, chart) that follows guidelines and format required by the practice setting.
d. Documents patient care consistent with guidelines and requirements of regulatory agencies and third-party payers.
e. Documents all necessary information in an organized manner that demonstrates sound clinical decision-making.
f. Produces documentation that is accurate, concise, timely and legible.
g. Utilizes terminology that is professionally and technically correct.
h. Documentation accurately describes care delivery that justifies physical therapy services.
i. Participates in quality improvement* review of documentation (chart audit, peer review, goals achievement).

MIDTERM COMMENTS: (Provide comments based on the performance dimensions including supervision/guidance*, quality*, complexity*, consistency*, and efficiency*.)

FINAL COMMENTS: (Provide comments based on the performance dimensions including supervision/guidance*, quality*, complexity*, consistency*, and efficiency*.)

Rate this student's clinical performance based on the sample behaviors and comments above:

Beginning Performance	Advanced Beginner Performance	Intermediate Performance	Advanced Intermediate Performance	Entry-level Performance	Beyond Entry-level Performance

Significant Concerns: If performance on this criterion is unacceptable, check the box and call the ACCE/DCE.

☎ **Midterm** ☐ ☎ **Final** ☐

PATIENT MANAGEMENT

OUTCOMES ASSESSMENT*

16. Collects and analyzes data from selected outcome measures in a manner that supports accurate analysis of individual patient and group outcomes.*

SAMPLE BEHAVIORS

a. Applies, interprets, and reports results of standardized assessments throughout a patient's episode of care.
b. Assesses and responds to patient and family satisfaction with delivery of physical therapy care.
c. Seeks information regarding quality of care rendered by self and others under clinical supervision.
d. Evaluates and uses published studies related to outcomes effectiveness.
e. Selects, administers, and evaluates valid and reliable outcome measures for patient groups.
f. Assesses the patient's response to intervention in practical terms.
g. Evaluates whether functional goals from the plan of care have been met.
h. Participates in quality/performance improvement programs (program evaluation, utilization of services, patient satisfaction).

MIDTERM COMMENTS: (Provide comments based on the performance dimensions including supervision/guidance*, quality*, complexity*, consistency*, and efficiency*.)

FINAL COMMENTS: (Provide comments based on the performance dimensions including supervision/guidance*, quality*, complexity*, consistency*, and efficiency*.)

Rate this student's clinical performance based on the sample behaviors and comments above:

Beginning Performance	Advanced Beginner Performance	Intermediate Performance	Advanced Intermediate Performance	Entry-level Performance	Beyond Entry-level Performance

Significant Concerns: If performance on this criterion is unacceptable, check the box and call the ACCE/DCE.

☏ **Midterm** ☐ ☏ **Final** ☐

PATIENT MANAGEMENT

FINANCIAL RESOURCES

17. Participates in the financial management (budgeting, billing and reimbursement, time, space, equipment, marketing, public relations) of the physical therapy service consistent with regulatory, legal, and facility guidelines.

SAMPLE BEHAVIORS

a. Schedules patients, equipment, and space.
b. Coordinates physical therapy with other services to facilitate efficient and effective patient care.
c. Sets priorities for the use of resources to maximize patient and facility outcomes.
d. Uses time effectively.
e. Adheres to or accommodates unexpected changes in the patient's schedule and facility's requirements.
f. Provides recommendations for equipment and supply needs.
g. Submits billing charges on time.
h. Adheres to reimbursement guidelines established by regulatory agencies, payers, and the facility.
i. Requests and obtains authorization for clinically necessary reimbursable visits.
j. Utilizes accurate documentation, coding, and billing to support request for reimbursement.
k. Negotiates with reimbursement entities for changes in individual patient services.
l. Utilizes the facility's information technology effectively.
m. Functions within the organizational structure of the practice setting.
n. Implements risk-management strategies (ie, prevention of injury, infection control, etc).
o. Markets services to customers (eg, physicians, corporate clients*, general public).
p. Promotes the profession of physical therapy.
q. Participates in special events organized in the practice setting related to patients and care delivery.
r. Develops and implements quality improvement plans (productivity, length of stay, referral patterns, and reimbursement trends).

MIDTERM COMMENTS: (Provide comments based on the performance dimensions including supervision/guidance*, quality*, complexity*, consistency*, and efficiency*.)

FINAL COMMENTS: (Provide comments based on the performance dimensions including supervision/guidance*, quality*, complexity*, consistency*, and efficiency*.)

Rate this student's clinical performance based on the sample behaviors and comments above:

Beginning Performance	Advanced Beginner Performance	Intermediate Performance	Advanced Intermediate Performance	Entry-level Performance	Beyond Entry-level Performance

Significant Concerns: If performance on this criterion is unacceptable, check the box and call the ACCE/DCE.

☎ **Midterm** ☐ ☎ **Final** ☐

PATIENT MANAGEMENT

DIRECTION AND SUPERVISION OF PERSONNEL

18. Directs and supervises personnel to meet patient's goals and expected outcomes according to legal standards and ethical guidelines.

SAMPLE BEHAVIORS

a. Determines those physical therapy services that can be directed to other support personnel according to jurisdictional law, practice guidelines, policies, codes of ethics, and facility policies.
b. Applies time-management principles to supervision and patient care.
c. Informs the patient of the rationale for and decision to direct aspects of physical therapy services to support personnel (eg, secretary, volunteers, PT Aides, Physical Therapist Assistants).
d. Determines the amount of instruction necessary for personnel to perform directed tasks.
e. Provides instruction to personnel in the performance of directed tasks.
f. Supervises those physical therapy services directed to physical therapist assistants* and other support personnel according to jurisdictional law, practice guidelines, policies, codes of ethics, and facility policies.
g. Monitors the outcomes of patients receiving physical therapy services delivered by other support personnel.
h. Demonstrates effective interpersonal skills including regular feedback in supervising directed support personnel.
i. Demonstrates respect for the contributions of other support personnel.
j. Directs documentation to physical therapist assistants that is based on the plan of care that is within the physical therapist assistant's ability and consistent with jurisdictional law, practice guidelines, policies, codes of ethics, and facility policies.
k. Reviews, in conjunction with the clinical instructor, physical therapist assistant documentation for clarity and accuracy.

MIDTERM COMMENTS: (Provide comments based on the performance dimensions including supervision/guidance*, quality*, complexity*, consistency*, and efficiency*.)

FINAL COMMENTS: (Provide comments based on the performance dimensions including supervision/guidance*, quality*, complexity*, consistency*, and efficiency*.)

Rate this student's clinical performance based on the sample behaviors and comments above:

Beginning Performance	Advanced Beginner Performance	Intermediate Performance	Advanced Intermediate Performance	Entry-level Performance	Beyond Entry-level Performance

Significant Concerns: **If performance on this criterion is unacceptable, check the box and call the ACCE/DCE.**

☎ **Midterm** ☐ ☎ **Final** ☐

SUMMATIVE COMMENTS

Given this student's level of academic and clinical preparation and the objectives for this clinical experience, identify strengths and areas for further development. If this is the student's final clinical experience, comment on the student's readiness to practice as a physical therapist.

AREAS OF STRENGTH

Midterm:

Final:

AREAS FOR FURTHER DEVELOPMENT

Midterm:

Final:

OTHER COMMENTS

Midterm:

Final:

RECOMMENDATIONS

Midterm:

Final:

EVALUATION SIGNATURES

MIDTERM EVALUATION

For the Student
I, the student, have read and understood the disclaimer (page 4) and directions (pages 5-13) for the PT CPI. I have completed the on-line training (website) prior to using this instrument and completed the PT CPI midterm self-assessment according to the training and directions. I have also read, reviewed, and discussed my completed performance evaluation with the clinical instructor(s) who evaluated my performance.

Signature of Student — Date

Name of Academic Institution

For the Evaluator(s)
I/We, the evaluator(s), have read and understood the disclaimer (page 4) and directions (pages 5-13) for the PT CPI. I/We have completed the on-line training (website) prior to using this instrument. I/We have completed this instrument, as the evaluator(s) according to the training and directions for the PT CPI. I/We have prepared, reviewed, and discussed the midterm completed PT CPI with the student with respect to his/her clinical performance.

Evaluator Name (1) (Print) — Position/title

Signature of Evaluator (1) — Date

Evaluator Name (2) (Print) — Position/Title

Signature of Evaluator (2) — Date

CCCE Signature — Date

FINAL EVALUATION

For the Student

I, the student, have read and understood the disclaimer (page 4) and directions (pages 5-13) for the PT CPI. I have completed the on-line training (website) prior to using this instrument and completed the PT CPI final self-assessment according to the training and directions. I have also read, reviewed, and discussed my completed performance evaluation with the clinical instructor(s) who evaluated my performance.

______________________________ ______________________

Signature of Student Date

____

Name of Academic Institution

For the Evaluator(s)

I/We, the evaluator(s), have read and understood the disclaimer (page 4) and directions (pages 5-13) for the PT CPI. I/We have completed the on-line training (website) prior to using this instrument. I/We have completed this instrument, as the evaluator(s) according to the training and directions for the PT CPI. I/We have prepared, reviewed, and discussed the final completed PT CPI with the student with respect to his/her clinical performance.

______________________________ ______________________

Evaluator Name (1) (Print) Position/title

______________________________ ______________________

Signature of Evaluator (1) Date

______________________________ ______________________

Evaluator Name (2) (Print) Position/Title

______________________________ ______________________

Signature of Evaluator (2) Date

______________________________ ______________________

CCCE Signature Date

GLOSSARY

Academic coordinator/Director of clinical education (ACCE/DCE): Individual who is responsible for managing and coordinating the clinical education program at the academic institution, including facilitating clinical site and clinical faculty development. This person also is responsible for the academic program and student performance, and maintaining current information on clinical sites.

Accountability: Active acceptance of responsibility for the diverse roles, obligations, and actions of the physical therapist including self-regulation and other behaviors that positively influence patient/client outcomes, the profession, and the health needs of society. (*Professionalism in Physical Therapy: Core Values,* August 2003.)

Adaptive devices: A variety of implements or equipment used to aid patients/clients in performing movements, tasks, or activities. Adaptive devices include raised toilet seats, seating systems, environmental controls, and other devices.

Advanced beginner performance: A student who requires clinical supervision 75% – 90% of the time with simple patients, and 100% of the time with complex patients. At this level, the student demonstrates developing proficiency with simple tasks (eg, medical record review, goniometry, muscle testing, and simple interventions) but is unable to perform skilled examinations, interventions, and clinical reasoning skills. The student may begin to share a caseload with the clinical instructor.

Advanced intermediate performance: A student who requires clinical supervision less than 25% of the time with new or complex patients and is independent with simple patients. At this level, the student is proficient in simple tasks and requires only occasional cueing for skilled examinations, interventions, and clinical reasoning. The student is capable of maintaining 75% of a full-time physical therapist's caseload.

Altruism: The primary regard for or devotion to the interest of patients/clients, thus assuming the fiduciary responsibility of placing the needs of the patient/client ahead of the physical therapist's self interest. *Professionalism in Physical Therapy: Core Values*, August 2003.)

Assessment: The measurement or quantification of a variable or the placement of a value on something. Assessment should not be confused with *examination* or *evaluation*.

Beginning performance: A student who requires close clinical supervision 100% of the time with constant monitoring and feedback, even with simple patients. At this level, performance is inconsistent and clinical reasoning is performed in an inefficient manner. Performance reflects little or no experience. The student does not carry a caseload.

Beyond entry-level performance: A student who is capable of functioning without clinical supervision with simple, highly complex patients, and is able to function in unfamiliar or ambiguous situations. Student is capable of supervising others. At this level, the student is consistently proficient at highly skilled examinations, interventions, and clinical reasoning, and is a capable of serving as a consultant or resource for others. Student is capable of maintaining 100% of a full-time physical therapist's caseload, seeks to assist others where needed. The student willingly assumes a leadership role for managing more difficult or complex cases. Actively contributes to the enhancement of the clinical facility with an expansive view of physical therapy practice and the profession.

Caring: The concern, empathy, and consideration for the needs and values of others. (*Professionalism in Physical Therapy: Core Values*, August 2003.)

Caregiver: One who provides care, often used to describe a person other than a health care professional.

Case management: The coordination of patient care or client activities.

Center Coordinator of Clinical Education: Individual who administers, manages, and coordinates CI assignments and learning activities for students during their clinical education experiences. In addition, this person determines the readiness of persons to serve as clinical instructors for students, supervises clinical instructors in the delivery of clinical education experiences, communicates with the academic program regarding student performance, and provides essential information about the clinical education program to physical therapy programs.

Client: An individual who is not necessarily sick or injured but who can benefit from a physical therapist=s consultation, professional advice, or services. A client also is a business, a school system, or other entity that may benefit from specific recommendations from a physical therapist.

Clinical decision making (CDM): Interactive model in which hypotheses are generated early in an encounter based on initial cures drawn from observation of the patient or client, a letter of referral, the medical record, or other resources.

Clinical education experiences: These experiences comprise all of the formal and practical "real-life" learning experiences provided for students to apply classroom knowledge and skills in the clinical environment. Experiences would include those of short and long duration (eg, part-time, full-time, internships) and those that provide a variety of learning experiences (eg, rotations on different units within the same practice setting, rotations between different practice settings within the same health care system) to include comprehensive care of patients across the life span and related activities.

Clinical indications: The patient factors (eg, symptoms, impairments, deficits) that suggest that a particular kind of care (examination, intervention) would be appropriate.

Clinical instructor (CI): Individual at the clinical education site who directly instructs and supervises students during their clinical learning experiences. CIs are responsible for facilitating clinical learning experiences and assessing students' performance in cognitive, psychomotor, and affective domains as related to entry-level clinical practice and academic and clinical performance expectations. (Syn: *clinical teacher, clinical tutor, and clinical supervisor.*)

Clinical reasoning: A systematic process used to assist students and practitioners in inferring or drawing conclusions about patient/client care under various situations and conditions.

Cognitive: Characterized by awareness, reasoning, and judgment.

Communication: A process by which information is exchanged between individuals through a common system of symbols, signs, or behavior.

Compassion: The desire to identify with or sense something of another's experience; a precursor of caring. (*Professionalism in Physical Therapy: Core Values*, August 2003.)

Competence: The possession, application, and evaluation of requisite professional knowledge, skills, and abilities to meet or exceed the performance standards, based on the physical therapist's roles and responsibilities, within the context of public health, welfare, and safety.

Competency: A significant, skillful, work-related activity that is performed efficiently, effectively, fluidly, and in a coordinated manner.

Complexity: Multiple requirements of the tasks or environment (eg, simple, complex), or patient (see Complex patient). The complexity of the tasks or environment can be altered by controlling the number and types of elements to be considered in the performance, including patients, equipment, issues, etc. As a student progresses through clinical education experiences, the complexity of tasks/environment should increase, with fewer elements controlled by the CI.

Complex patient: Refers to patients presenting with multiple co-morbidities, multi-system involvement, needs for extensive equipment, multiple lines, cognitive impairments, and multifaceted psychosocial needs. As a student progresses through clinical education experiences, the student should be able to manage patients with increasingly more complex conditions with fewer elements or interventions controlled by the CI.

Conflict management: The act, manner, or practice of handling or controlling the impact of disagreement, controversy, or opposition; may or may not involve resolution of the conflict.

Consistency: The frequency of occurrences of desired behaviors related to the performance criterion (eg, infrequently, occasionally, and routinely). As a student progresses through clinical education experiences, consistency of quality performance is expected to progress from infrequently to routinely.

Consultation: The rendering of professional or expert opinion or advice by a physical therapist. The consulting physical therapist applies highly specialized knowledge and skills to identify problems, recommend solutions, or produce a specified outcome or product in a given amount of time. (*Guide to Physical Therapist Practice*. Rev 2nd Ed. Alexandria, Va: American Physical Therapy Association; 2003.)

Consumer: One who acquires, uses, or purchases goods or services; any actual or potential recipient of health care.

Cost-effectiveness: Economically worthwhile in terms of what is achieved for the amount of money spent; tangible benefits in relation to expenditures.

Critical inquiry: The process of applying the principles of scientific methods to read and interpret professional literature, participate in research activities, and analyze patient care outcomes, new concepts, and findings.

Cultural awareness: Refers to the basic idea that behavior and ways of thinking and perceiving are culturally conditioned rather than universal aspects of human nature. (Pusch MD, ed. *Multicultural Education*. Yarmouth, Maine: Intercultural Press Inc; 1999.)

Cultural competence: Cultural and linguistic competence is a set of congruent behaviors, attitudes and policies that come together in a system, agency or among professionals that enables effective work in cross-cultural situations. "Culture" refers to integrated patterns of human behavior that include the language, thoughts, communications, actions, customs, beliefs, values, and institutions of racial, ethnic, religious or social groups. "Competence" implies having the capacity to function effectively as an individual and an organization within the context of the cultural beliefs, behaviors and needs presented by consumers and their communities. (Working definition adapted from *Assuring Cultural Competence in Health Care: Recommendations for National Standards and an Outcomes-Focused Research Agenda*, Office of Minority Health, Public Health Service, U S Department of Health and Human Services; 1999.

Cultural sensitivity: Awareness of cultural variables that may affect assessment and treatment. (Paniagua FA. *Assessing and Treating Culturally Diverse Clients*. Thousand Oaks, Calif: Sage Publications; 1994.)

Diagnosis: Diagnosis is both a process and a label. The diagnostic process performed by the physical therapist includes integrating and evaluating data that are obtained during the examination to describe the patient/client condition in terms that will guide the prognosis, the plan of care, and intervention strategies. Physical therapists use diagnostic labels that identify the impact of a condition on function at the level of the system (especially the movement system) and at the level of the whole person. (*Guide to Physical Therapist Practice*. Rev 2nd Ed. Alexandria, Va: American Physical Therapy Association; 2003.)

Diagnostic process: The evaluation of information obtained from the patient examination organized into clusters, syndromes, or categories.

Differential diagnosis: The determination of which one of two or more different disorders or conditions is applicable to a patient or client.

Direct access: Practice mode in which physical therapists examine, evaluate, diagnose, and provide interventions to patients/clients without a referral from a gatekeeper, usually the physician.

Disability: The inability to perform or a limitation in the performance of actions, tasks, and activities usually expected in specific social roles that are customary for the individual or expected for the person's status or role in a specific sociocultural context and physical environment. (*Guide to Physical Therapist Practice*. Rev 2nd Ed. Alexandria, Va: American Physical Therapy Association; 2003.)

Disease: A pathological condition or abnormal entity with a characteristic group of signs and symptoms affecting the body and with known or unknown etiology. (*Guide to Physical Therapist Practice*. Rev 2nd Ed. Alexandria, Va: American Physical Therapy Association; 2003.)

Discharge: The process of ending physical therapy services that have been provided during a single episode of care, when the anticipated goals and expected outcomes have been achieved. Discharge does not occur with a transfer (that is, when the patient is moved from one site to another site within the same setting or across setting during a single episode of care). (*Guide to Physical Therapist Practice*. Rev 2nd Ed. Alexandria, Va: American Physical Therapy Association; 2003.)

Documentation: All written forms of communication provided related to the delivery of patient care, to include written correspondence, electronic record keeping, and word processing.

Dysfunction: Disturbance, impairment, or abnormality of function of an organ. (*Guide to Physical Therapist Practice*. Rev 2nd Ed. Alexandria, Va: American Physical Therapy Association; 2003.)

Education: Knowledge or skill obtained or developed by a learning process; a process designed to change behavior by formal instruction and/or supervised practice, which includes teaching, training, information sharing, and specific instructions.

Efficiency: The ability to perform in a cost-effective and timely manner (eg, inefficient/slow, efficient/timely). As the student progresses though clinical education experiences, efficiency should progress from a high expenditure of time and effort to economical and timely.

Empathy: The action of understanding, being aware of, being sensitive to, and vicariously experiencing the feelings, thoughts, and experience of another of either the past or present without having the feelings, thoughts, and experience fully communicated in an objectively explicit manner.

Entry-level performance: A student who is capable of functioning without guidance or clinical supervision with simple or complex patients. Consults with others and resolves unfamiliar or ambiguous situations. At this level, the student is consistently proficient and skilled in simple and complex tasks for skilled examinations, interventions, and clinical reasoning. The student is capable of maintaining 100% of a full-time physical therapist's caseload in a cost effective manner.

Episode of physical therapy prevention: A series of occasional, clinical, educational, and administrative services related to primary prevention, wellness, health promotion, and to the preservation of optimal function. Prevention services and programs that promote health, wellness, and fitness are a vital part of the practice of physical therapy. No defined number or range of number of visits is established for this type of episode. (*Guide to Physical Therapist Practice*. Rev 2nd Ed. Alexandria, Va: American Physical Therapy Association; 2003.)

Evaluation: A dynamic process in which the physical therapist makes clinical judgments based on data gathered during the examination. No defined number or range of number of visits is established for this type of episode. (*Guide to Physical Therapist Practice*. Rev 2nd Ed. Alexandria, Va: American Physical Therapy Association; 2003.)

Evidenced-based practice: Integration of the best possible research evidence with clinical expertise and patient values, to optimize patient/client outcomes and quality of life to achieve the highest level of excellence in clinical practice. (Sackett DL, Haynes RB, Guyatt GH, Tugwell P. *Clinical Epidemiology: A Basic Science for Clinical Medicine*. 2nd ed. Boston: Little, Brown and Company; 1991:1.) Evidence includes randomized or nonrandomized controlled trials, testimony or theory, meta-analysis, case reports and anecdotes, observational studies, narrative review articles, case series in decision making for clinical practice and policy, effectiveness research for guidelines development, patient outcomes research, and coverage decisions by health care plans.

Examination: A comprehensive and specific testing process performed by a physical therapist that leads to diagnostic classification or, as appropriate, to a referral to another practitioner. The examination has three components: the patient/client history, the systems reviews, and tests and measures. (*Guide to Physical Therapist Practice*. Rev 2nd Ed. Alexandria, Va: American Physical Therapy Association; 2003.)

Excellence: Excellence is physical therapy practice that consistently uses current knowledge and theory while understanding personal limits, integrates judgment and the patient/client perspective, embraces advancement, challenges mediocrity, and works toward development of new knowledge. (*Professionalism in Physical Therapy: Core Values,* August 2003.)

Fiscal management: An ability to identify the fiscal needs of a unit and to manage available fiscal resources to maximize the benefits and minimize constraints.

Fitness: A dynamic physical state—comprising cardiovascular/pulmonary endurance; muscle strength, power, endurance, and flexibility; relaxation; and body composition—that allows optimal and efficient performance of daily and leisure activities. (*Guide to Physical Therapist Practice*. Rev 2nd Ed. Alexandria, Va: American Physical Therapy Association; 2003.)

Function: The special, normal, or proper action of any part or organ; an activity identified by an individual as essential to support physical and psychological well-being as well as to create a personal sense of meaningful living; the action specifically for which a person or thing is fitted or employed; an act, process, or series of processes that serve a purpose; to perform an activity or to work properly or normally.

Functional limitation: A restriction of the ability to perform a physical action, activity, or task in a typically expected, efficient, or competent manner. (*Guide to Physical Therapist Practice*. Rev 2nd Ed. Alexandria, Va: American Physical Therapy Association; 2003.)

Functional outcomes: The desired result of an act, process, or intervention that serves a purpose (eg, improvement in a patient's ability to engage in activities identified by the individual as essential to support physical or psychological well-being).

Goals: The intended results of patient/client management. Goals indicate changes in impairment, functional limitations, and disabilities and changes in health, wellness, and fitness needs that are expected as a result of implementing the plan of care. Goals should be measurable and time limited (if required, goals may be expressed as short-term and long-term goals.) (*Guide to Physical Therapist Practice*. Rev 2nd Ed. Alexandria, Va: American Physical Therapy Association; 2003.)

Guide to Physical Therapist Practice: Document that describes the scope of practice of physical therapy and assists physical therapists in patient/client management. Specifically, the *Guide* is designed to help physical therapists: 1) enhance quality of care, 2) improve patient/client satisfaction, 3) promote appropriate utilization of health care services, 4) increase efficiency and reduce unwarranted variation in the provision of services, and 5) promote cost reduction through prevention and wellness initiatives. The *Guide* also provides a framework for physical therapist clinicians and researchers as they refine outcomes data collection and analysis and develop questions for clinical research. (*Guide to Physical Therapist Practice*. Rev 2nd Ed. Alexandria, Va: American Physical Therapy Association; 2003.)

Health care provider: A person or organization offering health services directly to patients or clients.

Health promotion: The combination of educational and environmental supports for actions and conditions of living conducive to health. The purpose of health promotion is to enable people to gain greater control over the determinants of their own health. (Green LW, Kreuter MW. *Health Promotion Planning.* 2nd ed. Mountain View, Calif: Mayfield Publishers; 1991:4.)

Health status: The level of an individual's physical, mental, affective, and social function: health status is an element of well-being.

History: An account of past and present health status that includes the identification of complaints and provides the initial source of information about the patient. The history also suggests the patient=s ability to benefit from physical therapy services.

Personnel management: Selection, training, supervision, and deployment of appropriately qualified persons for specific tasks/functions.

Impairment: A loss or abnormality of physiological, psychological, or anatomical structure or function. (*Guide to Physical Therapist Practice*. Rev 2nd Ed. Alexandria, Va: American Physical Therapy Association; 2003.)

Integrity: *S*teadfast adherence to high ethical principles or professional standards; truthfulness, fairness, doing what you say you will do, and "speaking forth" about why you do what you do. (*Professionalism in Physical Therapy: Core Values,* August 2003.)

Intermediate clinical performance: A student who requires clinical supervision less than 50% of the time with simple patients, and 75% of the time with complex patients. At this level, the student is proficient with simple tasks and is developing the ability to perform skilled examinations, interventions, and clinical reasoning. The student is capable of maintaining 50% of a full-time physical therapist's caseload.

Intervention: The purposeful interaction of the physical therapist with the patient/client, and, when appropriate, with other individuals involved in patient/client care, using various physical therapy procedures and techniques to produce changes in the condition. (*Guide to Physical Therapist Practice*. Rev 2nd Ed. Alexandria, Va: American Physical Therapy Association; 2003.)

Manual therapy techniques: Skilled hand movements intended to improve tissue extensibility; increase range of motion; induce relaxation; mobilize or manipulate soft tissue and joints; modulate pain; and reduce soft tissue swelling, inflammation, or restriction. (*Guide to Physical Therapist Practice*. Rev 2nd Ed. Alexandria, Va: American Physical Therapy Association; 2003.)

Mobilization/manipulation: A manual therapy technique comprising a continuum of skilled passive movements to the joints and/or related soft tissues that are applied at varying speeds and amplitudes, including a small amplitude/high velocity therapeutic movement. (*Guide to Physical Therapist Practice*. Rev 2nd Ed. Alexandria, Va: American Physical Therapy Association; 2003.)

Multicultural/multilingual: Characteristics of populations defined by changes in the demographic patterns of consumers.

Negotiation: The act or procedure of treating another or others in order to come to terms or reach an agreement.

Objective: A measurable behavioral statement of an expected response or outcome; something worked toward or striven for; a statement of direction or desired achievement that guides actions and activities.

Outcomes assessment of the individual: Performed by the physical therapist and is a measure (or measures) of the intended results of patient/client management, including changes in impairments, functional limitations, and disabilities and the changes in health, wellness, and fitness needs that are

expected as the results of implementing the plan of care. The expected outcomes in the plan should be measurable and time limited.

Outcomes assessment of groups of patients/clients: Performed by the physical therapist and is a measure [or measures] of physical therapy care to groups of patients/clients including changes in impairments, functional limitations, and disabilities and the changes in health, wellness, and fitness needs that are expected as the results of that physical therapy.

Outcomes analysis: A systematic examination of patient/client outcomes in relation to selected patient/client variables (eg, age, sex, diagnosis, interventions performed); outcomes analysis may be used in quality assessment, economic analysis of practice, and other processes.

Patients: Individuals who are the recipients of physical therapy and direct interventions.

Patient/client management model:

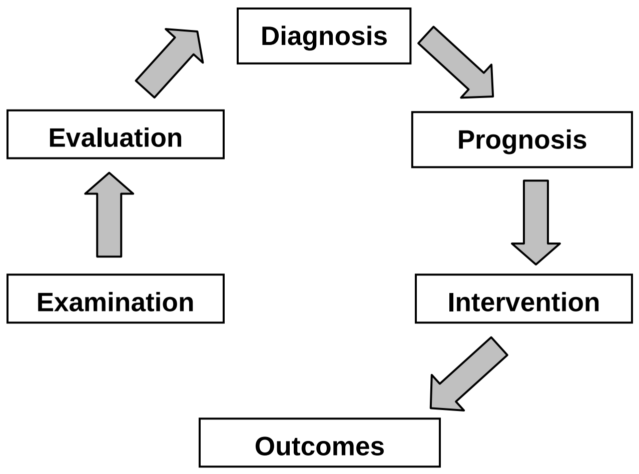

(Adapted from the *Guide to Physical Therapist Practice*. Rev 2nd Ed. Alexandria, Va: American Physical Therapy Association; 2003.)

Performance criterion: A description of outcome knowledge, skills, and behaviors that define the expected performance of students. When criteria are taken in aggregate, they describe the expected performance of the graduate upon entry into the practice of physical therapy.

Physical function: Fundamental components of health status describing the state of those sensory and motor skills necessary for mobility, work, and recreation.

Physical therapist: A licensed health care professional who offers services designed to preserve, develop, and restore maximum physical function.

Physical therapist assistant: An educated health care provider who performs physical therapy procedures and related tasks that have been selected and delegated by the supervising physical therapist.

Plan of care: (Statements that specify the anticipated goals and the expected outcomes, predicted level of optimal improvement, specific interventions to be used, and proposed duration and frequency of the interventions that are required to reach the goals and outcomes. The plan of care includes the anticipated discharge plans. (*Guide to Physical Therapist Practice*. Rev 2nd Ed. Alexandria, Va: American Physical Therapy Association; 2003.)

Practice management: The coordination, promotion, and resource (financial and human) management of practice that follows regulatory and legal guidelines.

Practitioner of choice: Consumers choose the most appropriate health care provider for the diagnosis, intervention, or prevention of an impairment, functional limitation, or disability.

Presenting problem: The specific dysfunction that causes an individual to seek attention or intervention (ie, chief complaint).

Prevention: Activities that are directed toward 1) achieving and restoring optimal functional capacity, 2) minimizing impairments, functional limitations, and disabilities, 3) maintaining health (thereby preventing further deterioration or future illness), 4) creating appropriate environmental adaptations to enhance independent function. *Primary prevention:* Prevention of disease in a susceptible or potentially susceptible population through such specific measures as general health promotion efforts. *Secondary prevention:* Efforts to decrease the duration of illness, severity of diseases, and sequelae through early diagnosis and prompt intervention. *Tertiary prevention:* Efforts to limit the degree of disability and promote rehabilitation and restoration of function in patients/clients with chronic and irreversible diseases. (*Guide to Physical Therapist Practice*. Rev 2nd Ed. Alexandria, Va: American Physical Therapy Association; 2003.)

Professional duty: Professional duty is the commitment to meeting one's obligations to provide effective physical therapy services to individual patients/clients, to serve the profession, and to positively influence the health of society. (*Professionalism in Physical Therapy: Core Values*, August 2003.)

Professionalism: The conduct, aims, or qualities that characterize or mark a profession or a professional person; A systematic and integrated set of core values that through assessment, critical reflection, and change, guides the judgment, decisions, behaviors, and attitudes of the physical therapist, in relation to patients/ clients, other professionals, the public, and the profession. (APTA Consensus Conference to Develop Core Values in Physical Therapy, July 2002, Alexandria, Va)

Prognosis: The determination by the physical therapist of the predicted optimal level of improvement in function and the amount of time needed to reach that level. (*Guide to Physical Therapist Practice*. Rev 2nd Ed. Alexandria, Va: American Physical Therapy Association; 2003.)

Quality: The degree of skill or competence demonstrated (eg, limited skill, high skill), the relative effectiveness of the performance (eg, ineffective, highly effective), and the extent to which outcomes meet the desired goals. A continuum of quality might range from demonstration of limited skill and effectiveness to a highly skilled and highly effective performance.

Quality improvement (QI): A management technique to assess and improve internal operations. Quality improvement focuses on organizational systems rather than individual performance and seeks to continuously improve quality rather than reacting when certain baseline statistical thresholds are crossed. The process involves setting goals, implementing systematic changes, measuring outcomes, and making subsequent appropriate improvements. (www.tmci.org/other_resources/glossaryquality.html#quality)

Role: A behavior pattern that defines a person's social obligations and relationships with others (eg, father, husband, son).

Reexamination: The process of performing selected tests and measures after the initial examination to evaluate progress and to modify or redirect interventions. (*Guide to Physical Therapist Practice*. Rev 2nd Ed. Alexandria, Va: American Physical Therapy Association; 2003.)

Screening: Determining the need for further examination or consultation by a physical therapist or for referral to another health professional. (*Guide to Physical Therapist Practice*. Rev 2nd Ed. Alexandria, Va: American Physical Therapy Association; 2003.) (See also: *Cognitive screening*.)

Social responsibility: The promotion of a mutual trust between the physical therapist as a part of the profession and the larger public that necessitates responding to societal needs for health and wellness. (*Professionalism in Physical Therapy: Core Values*, August 2003.)

Supervision/guidance: Level and extent of assistance required by the student to achieve clinical performance at entry-level. As a student progresses through clinical education experiences, the degree of monitoring needed is expected to progress from full-time monitoring/direct supervision or cuing for assistance to initiate, to independent performance with consultation. The degree of supervision and guidance may vary with the complexity of the patient or environment.

Technically competent: Correct performance of a skill.

Tests and measures: Specific standardized methods and techniques used to gather data about the patient/client after the history and systems review have been performed. (*Guide to Physical Therapist Practice*. Rev 2nd Ed*.* Alexandria, Va: American Physical Therapy Association; 2003.)

Treatment: The sum of all interventions provided by the physical therapist to a patient/client during an episode of care. (*Guide to Physical Therapist Practice*. Rev 2nd Ed*.* Alexandria, Va: American Physical Therapy Association; 2003.)

Wellness: An active process of becoming aware of and making choices toward a more successful existence. (National Wellness Organization. *A Definition of Wellness*. Stevens Point, Wis: National Wellness Institute Inc; 2003.)

APPENDIX A
EXAMPLE: COMPLETED ITEM FOR FINAL EXPERIENCE (Competent)

EXAMINATION*

9. Performs a physical therapy patient examination* using evidenced-based* test and measures.

SAMPLE BEHAVIORS

a) Obtains a history from patients and other sources as part of the examination.*
b) Utilizes information from history and other data (eg, laboratory, diagnostic tests and pharmacological information) to formulate initial hypothesis and prioritize selection of test and measures.
c) Performs systems review.
d) Selects evidence-based tests and measures* that are relevant to the history, chief complaint, and screening.
   Tests and measures* (listed alphabetically) include, but are not limited to, the following: a) aerobic capacity, b) anthropometric characteristics, c) arousal, mentation, and cognition, d) assistive and adaptive devices*, e) community and work (job, school, or play) reintegration, f) cranial nerve integrity, g) environmental, home, and work barriers, h) ergonomics and body mechanics, i) gait, assisted locomotion, and balance, j) integumentary integrity, k) joint integrity and mobility, l) motor function*, m) muscle performance (including strength, power, and endurance), n) neuromotor development and sensory integration, o) orthotic, protective, and supportive devices, p) pain, q) posture, r) prosthetic requirements, s) range of motion, t) reflex integrity, u) self-care and home management (including activities of daily living and instrumental activities of daily living), v) sensory integration (including proprioception and kinesthesia), and w) ventilation, respiration, and circulation.
e) Conducts tests and measures accurately and proficiently.
f) Sequences tests and measures in a logical manner to optimize efficiency*.
g) Adjusts tests and measures according to patient's response.
h) Performs regular re-examinations of patient status.
i) Performs an examination using evidence based test and measures.

MIDTERM COMMENTS: (Provide comments based on the performance dimensions including *supervision/ guidance, quality, complexity, consistency, and efficiency*.)

This student requires guidance 25% of the time in selecting appropriate examination methods based on the patient's history and initial screening. Examinations are performed consistently, accurately, thoroughly, and skillfully. She almost always is able to complete examinations in the time allotted, except for patients with the most complex conditions. She manages a 75% caseload of the PT with some difficulty and requires assistance in completing the examination for a patient with a complex condition of dementia and multiple diagnoses. Overall she has achieved a level of performance consistent with advanced intermediate performance for this criterion and continues to improve in all areas.

FINAL COMMENTS: (Provide comments based on the performance dimensions including *supervision/ guidance, quality, complexity, consistency, and efficiency**.)

This student requires no guidance in selecting appropriate examination methods for patients with complex conditions and with multiple diagnoses. Examinations are performed consistently and skillfully. She consistently selects all appropriate examination methods based on the patient's history and initial screening. She consistently completes examinations in the time allotted and manages a 100% caseload of the PT. She is able to examine a number of patients with complex conditions and with multiple diagnoses with only minimal input from the CI. Overall this student has improved across all performance dimensions to achieve entry-level clinical performance.

Rate this student's clinical performance based on the sample behaviors and comments above:

Beginning Performance	Advanced Beginner Performance	Intermediate Performance	Advanced Intermediate Performance	Entry-level Performance	Beyond Entry-level Performance
			M	F	

Significant Concerns: If performance on this criterion is unacceptable, check the box and call the ACCE/DCE.

☎ **Midterm** ☐ ☎ **Final** ☐

APPENDIX A
EXAMPLE: COMPLETED ITEM FOR FINAL EXPERIENCE (Not Competent)

EXAMINATION*

9. Performs a physical therapy patient examination* using evidenced-based* test and measures.

SAMPLE BEHAVIORS

e) Obtains a history from patients and other sources as part of the examination.
f) Utilizes information from history and other data (eg, laboratory, diagnostic tests and pharmacological information) to formulate initial hypothesis and prioritize selection of test and measures.
g) Performs systems review.
h) Selects evidence-based tests and measures that are relevant to the history, chief complaint, and screening.
Tests and measures (listed alphabetically) include, but are not limited to, the following: a) aerobic capacity, b) anthropometric characteristics, c) arousal, mentation, and cognition, d) assistive and adaptive devices*, e) community and work (job, school, or play) reintegration, f) cranial nerve integrity, g) environmental, home, and work barriers, h) ergonomics and body mechanics, i) gait, assisted locomotion, and balance, j) integumentary integrity, k) joint integrity and mobility, l) motor function*, m) muscle performance (including strength, power, and endurance), n) neuromotor development and sensory integration, o) orthotic, protective, and supportive devices, p) pain, q) posture, r) prosthetic requirements, s) range of motion, t) reflex integrity, u) self-care and home management (including activities of daily living and instrumental activities of daily living), v) sensory integration (including proprioception and kinesthesia), and w) ventilation, respiration, and circulation.
j) Conducts tests and measures accurately and proficiently.
k) Sequences tests and measures in a logical manner to optimize efficiency*.
l) Adjusts tests and measures according to patient's response.
m) Performs regular re-examinations of patient status.
n) Performs an examination using evidence based test and measures.

MIDTERM COMMENTS: (Provide comments based on the performance dimensions including *supervision/ guidance, quality, complexity, consistency, and efficiency**.)

This student requires guidance 75% of the time to select relevant tests and measures and does not ask relevant background questions to identify tests and measures needed. Tests and measures selected are inappropriate for the patient's diagnosis and condition. When questioned, he is unable to explain why specific tests and measures were selected. He is not accurate in performing examination techniques (eg, fails to correctly align the goniometer, places patients in uncomfortable examination positions) and requires assistance when completing exams on all patients with complex conditions and with 75% of patients with simple conditions. He is unable to complete 60% of the exams in the time allotted and demonstrates difficulty across all performance dimensions for the final clinical experience.

FINAL COMMENTS: (Provide comments based on the performance dimensions including *supervision/guidance, quality, complexity, consistency, and efficiency**.)

This student requires guidance 50% of the time to select relevant tests and measures. He selects tests and measures that are appropriate for patients with simple conditions 50% of the time, however 50% of the time is unable to explain the tests and measures selected. Likewise, 50% of the time, he selects tests and measures that are inappropriate for the patient's diagnosis. He demonstrates 50% accuracy in performing the required examination techniques, including goniometry and requires assistance to complete examinations on 95% of patients with complex conditions and 50% of patients with simple conditions. He is unable to complete 50% of the exams in the time allotted. Although some limited improvement has been shown, performance across all performance dimensions for the final clinical experience is still in the advanced beginner performance interval, which is below expected performance of entry-level on this criterion for a final clinical experience.

Rate this student's clinical performance based on the sample behaviors and comments above:

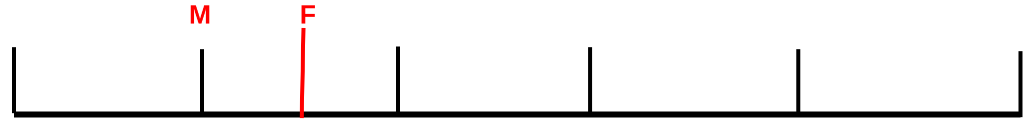

Beginning Performance	Advanced Beginner Performance	Intermediate Performance	Advanced Intermediate Performance	Entry-level Performance	Beyond Entry-level Performance

Significant Concerns: If performance on this criterion is unacceptable, check the box and call the ACCE/DCE.

☎ **Midterm** ☒ ☎ **Final** ☒

APPENDIX A
COMPLETED FOR INTERMEDIATE EXPERIENCE (COMPETENT)

EXAMINATION*

9. Performs a physical therapy patient examination* using evidenced-based* test and measures.

SAMPLE BEHAVIORS

i) Obtains a history from patients and other sources as part of the examination.
j) Utilizes information from history and other data (eg, laboratory, diagnostic tests and pharmacological information) to formulate initial hypothesis and prioritize selection of test and measures.
k) Performs systems review.
l) Selects evidence-based tests and measures that are relevant to the history, chief complaint, and screening.
  Tests and measures (listed alphabetically) include, but are not limited to, the following: a) aerobic capacity, b) anthropometric characteristics, c) arousal, mentation, and cognition, d) assistive and adaptive devices*, e) community and work (job, school, or play) reintegration, f) cranial nerve integrity, g) environmental, home, and work barriers, h) ergonomics and body mechanics, i) gait, assisted locomotion, and balance, j) integumentary integrity, k) joint integrity and mobility, l) motor function*, m) muscle performance (including strength, power, and endurance), n) neuromotor development and sensory integration, o) orthotic, protective, and supportive devices, p) pain, q) posture, r) prosthetic requirements, s) range of motion, t) reflex integrity, u) self-care and home management (including activities of daily living and instrumental activities of daily living), v) sensory integration (including proprioception and kinesthesia), and w) ventilation, respiration, and circulation.
o) Conducts tests and measures accurately and proficiently.
p) Sequences tests and measures in a logical manner to optimize efficiency*.
q) Adjusts tests and measures according to patient's response.
r) Performs regular re-examinations of patient status.
s) Performs an examination using evidence based test and measures.

MIDTERM COMMENTS: (Provide comments based on the performance dimensions including *supervision/ guidance, quality, complexity, consistency, and efficiency**.)

This student requires supervision for managing patients with simple conditions 50% of the time and managing patients with complex neurological conditions 95% of the time. He selects relevant examination methods for patients with simple conditions 85% of the time, however sometimes over tires patients during the examination. He requires limited assistance to perform examination methods accurately (sensory testing) and completes examinations in the time allotted most of the time. He carries a 25% caseload of the PT and is able to use good judgment in the selection and implementation of examinations for this level of clinical experience.

FINAL COMMENTS: (Provide comments based on the performance dimensions including *supervision/guidance, quality, complexity, consistency, and efficiency**.)

The student requires supervision for managing patients with simple conditions 25% of the time and managing patients with complex conditions 75% of the time. He selects relevant examination methods for patients with simple conditions 100% of the time and consistently monitors the patient's fatigue level during the examination. He performs complete and accurate examinations of patients with simple orthopedic conditions and is beginning to describe movement patterns in patients with complex neurological conditions. However, he continues to require frequent input to complete a neurological examination and is unable to consistently complete examinations in the time allotted. He carries a 50% caseload of the PT and has shown improvement in advancing from advanced beginner performance to intermediate performance for this second clinical experience.

Rate this student's clinical performance based on the sample behaviors and comments above:

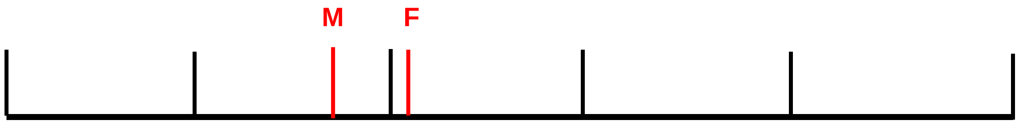

Beginning Performance | Advanced Beginner Performance | Intermediate Performance | Advanced Intermediate Performance | Entry-level Performance | Beyond Entry-level Performance

Significant Concerns: If performance on this criterion is unacceptable, check the box and call the ACCE/DCE.

☎ **Midterm** ☐ ☎ **Final** ☐

APPENDIX B
PT CPI Performance Criteria Matched with Evaluative Criteria for PT Programs

This table provides the physical therapist academic program with a mechanism to relate the performance criteria from the *Physical Therapist Clinical Performance Instrument* with the *Evaluative Criteria for Accreditation of Education Programs for the Preparation of Physical Therapists*.[1]

Evaluative Criteria for Accreditation of Physical Therapist Programs	Physical Therapist Clinical Performance Instrument Performance Criteria (PC)
**Accountability** (5.1-5.5)	**Accountability** (PC #3; 5.1-5.3)<br>**Professional Development** (PC #6; 5.4, 5.5)
**Altruism** (5.6, 5.7)	**Accountability** (PC #3; 5.6 and 5.7)
**Compassion/Caring** (5.8, 5.9)	**Professional Behavior** (PC #2; 5.8)<br>**Plan of Care** (PC #12, #13; 5.9)
**Integrity** (5.10)	**Professional Behavior** (PC #2; 5.10)
**Professional Duty** (5.11-5.16)	**Professional Behavior** (PC #2; 5.11, 5.15, 5.16)<br>**Professional Development** (PC #6, 5.12, 5.13, 5.14, 5.15)
**Communication** (5.17)	**Communication** (PC #4; 5.17**)**
**Cultural Competence** (5.18)	**Cultural Competence** (PC #5, 5.18)
**Clinical Reasoning** (5.19, 5.20)	**Clinical Reasoning** (PC #7; 5.19, 5.20)
**Evidenced-Based Practice** (5.21-5.25)	**Clinical Reasoning** (PC #7; 5.21, 5.22, 5.23)<br>**Professional Development** (PC #6; 5.24, 5.25)
**Education** (5.26)	**Educational Interventions** (PC #14; 5.26)
**Screening** (5.27)	**Screening** (PC #8; 5.27)
**Examination** (5.28-5.30)	**Examination** (PC #9; 5.28, 5.29, 5.30)
**Evaluation** (5.31)	**Evaluation** (PC #10; 5.31)
**Diagnosis** (5.32)	**Diagnosis and Prognosis** (PC #11; 5.32)
**Prognosis** (5.33)	**Diagnosis and Prognosis** (PC #11; 5.33)
**Plan of Care** (5.34-5.38)	**Plan of Care** (PC #12; 5.34, 5.35, 5.36, 5.37, 5.38)<br>**Safety** (PC #1; 5.35)
**Intervention** (5.39-5.44)	**Procedural Interventions** (PC #13; 5.39)<br>**Direction and Supervision of Personnel** (PC #18; 5.40)<br>**Educational Interventions** (PC #14; 5.41)<br>**Documentation** (PC #15; 5.42)<br>**Financial Resources** (PC #17; 5.43)<br>**Safety** (PC #1; 5.44)
**Outcomes Assessment** (5.45-5.49)	**Outcomes Assessment** (PC #16; 5.45, 5.46, 5.47, 5.48, 5.49)
**Prevention, Health Promotion, Fitness, and Wellness** (5.50-5.52)	**Procedural Interventions** (PC #13; 5.50, 5.52**)**<br>**Educational Interventions** (PC #14; 5.51, 5.52)
**Management in Care Delivery (**5.53-5.56)	**Screening** (PC #8; 5.53; 5.54, 5.55)<br>**Plan of Care** (PC #12; 5.55, 5.56 *[however not specifically stated as case management*])*<br>**Financial Resources** (PC #17; 5.55)
**Practice Management** (5.57-5.61)	**Financial Resources** (PC #17; 5.58, 5.60, 5.61)<br>**Direction and Supervision of Personnel** (PC #18; 5.57)<br>**Not included:** 5.59
**Consultation** (5.62)	**Screening** (PC #8; 5.62)<br>**Educational Interventions** (PC #14; 5.62)
**Social Responsibility and Advocacy** (5.63-5.66)	**Accountability** (PC #2; 5.63-5.66)

[1]*Evaluative Criteria for Accreditation of Education Programs for the Preparation of Physical Therapists.* Commission on Accreditation in Physical Therapy Education, APTA: Alexandria, VA; Adopted 2004; last revised 10/09; B29-B33.

APPENDIX C
DEFINITIONS OF PERFORMANCE DIMENSIONS AND RATING SCALE ANCHORS

CATEGORY	DEFINITIONS
	Performance Dimensions
**Supervision/ Guidance**	Level and extent of assistance required by the student to achieve entry-level performance.<br>▪ As a student progresses through clinical education experiences, the degree of supervision/guidance needed is expected to progress from 100% supervision to being capable of independent performance with consultation and may vary with the complexity of the patient or environment.
**Quality**	Degree of knowledge and skill proficiency demonstrated.<br>▪ As a student progresses through clinical education experiences, quality should range from demonstration of limited skill to a skilled performance.
**Complexity**	Number of elements that must be considered relative to the task, patient, and/or environment.<br>▪ As a student progresses through clinical education experiences, the level of complexity of tasks, patient management, and the environment should increase, with fewer elements being controlled by the CI.
**Consistency**	Frequency of occurrences of desired behaviors related to the performance criterion.<br>▪ As a student progresses through clinical education experiences, consistency of quality performance is expected to progress from infrequently to routinely.
**Efficiency**	Ability to perform in a cost-effective and timely manner.<br>▪ As the student progresses through clinical education experiences, efficiency should progress from a high expenditure of time and effort to economical and timely performance.
	Rating Scale Anchors
**Beginning performance**	• A student who requires close clinical supervision 100% of the time managing patients with constant monitoring and feedback, even with patients with simple conditions.<br>• At this level, performance is inconsistent and clinical reasoning* is performed in an inefficient manner.<br>• Performance reflects little or no experience.<br>• The student does not carry a caseload.
**Advanced beginner performance**	• A student who requires clinical supervision 75% – 90% of the time managing patients with simple conditions, and 100% of the time managing patients with complex conditions.<br>• At this level, the student demonstrates consistency in developing proficiency with simple tasks (eg, medical record review, goniometry, muscle testing, and simple interventions), but is unable to perform skilled examinations, interventions, and clinical reasoning skills.<br>• The student may begin to share a caseload with the clinical instructor.
**Intermediate performance**	• A student who requires clinical supervision less than 50% of the time managing patients with simple conditions, and 75% of the time managing patients with complex conditions.<br>• At this level, the student is proficient with simple tasks and is developing the ability to consistently perform skilled examinations, interventions, and clinical reasoning.<br>• The student is **capable of** maintaining 50% of a full-time physical therapist's caseload.
**Advanced intermediate performance**	• A student who requires clinical supervision less than 25% of the time managing new patients or patients with complex conditions and is independent managing patients with simple conditions.<br>• At this level, the student is consistent and proficient in simple tasks and requires only occasional cueing for skilled examinations, interventions, and clinical reasoning.<br>• The student is **capable of** maintaining 75% of a full-time physical therapist's caseload.
**Entry-level performance**	• A student who is **capable of** functioning without guidance or clinical supervision managing patients with simple or complex conditions.<br>• At this level, the student is consistently proficient and skilled in simple and complex tasks for skilled examinations, interventions, and clinical reasoning.<br>• Consults with others and resolves unfamiliar or ambiguous situations.<br>• The student is **capable of** maintaining 100% of a full-time physical therapist's caseload in a cost effective manner.
**Beyond entry-level performance**	• A student who is **capable of** functioning without clinical supervision or guidance in managing patients with simple or highly complex conditions, and is able to function in unfamiliar or ambiguous situations.<br>• At this level, the student is consistently proficient at highly skilled examinations, interventions, and clinical reasoning, and is a capable of serving as a consultant or resource for others.<br>• The student is **capable of** maintaining 100% of a full-time physical therapist's caseload and seeks to assist others where needed.<br>• The student is capable of supervising others.<br>• The student willingly assumes a leadership role* for managing patients with more difficult or complex conditions.
