# Supplementary material for: Validity of the formative physical therapy Student and Clinical Instructor Performance Assessment Instrument in the United States: a quasi-experimental, time-series study
Source: J Educ Eval Health Prof. 2025 Sep 26;22:26. doi: 10.3352/jeehp.2025.22.26 (PMC12688320; doi:10.3352/jeehp.2025.22.26)
Supplement: Supplementary file 4 — Supplement 3. SCIPAI internal structure analyses. [file jeehp-22-26-suppl3.docx]

**Supplement 3. SCIPAI internal structure analyses**

Split-half reliability, interitem correlations, and corrected item-total correlation analyses each suggest that the SCIPAI demonstrates acceptable internal structure evidence, with its items maintaining sufficient construct similarity and uniqueness.

**Analyses of CI Performance Items: Supervision, Feedback**

Spearman-Brown split-half reliability analyses of CI performance items of supervision and feedback indicate good internal consistency among student (r_sb_=.857) and CI (r_sb_=.841) ratings of CI performance.

**Analyses of Student Performance Items: Evaluation, Treatment, Communication, Professional behavior**

Average interitem correlations for student performance items as rated by CIs (r=.375) and students (r=.382) fall in the r range of .20-.40, suggesting an appropriate balance between item homogeneity and uniqueness (as detailed in Piedmont RL. Inter-item Correlations. In: Michalos, A.C. (eds) Encyclopedia of Quality of Life and Well-Being Research. 2014. Springer, Dordrecht. https://doi.org/10.1007/978-94-007-0753-5_1493).

Corrected item-total correlations indicated very good discrimination between high and low scores on CI and student ratings of student performance items of evaluation, treatment, and communication; and indicated low discrimination for professional behavior, according to criterion values.

**Supplement 3 Table A. Correlated Item-Total Correlation**

| **Final SCIPAI Item** | **Clinical Instructor as Rater Corrected Item Total Correlation** (N=607) | **Student as Rater Corrected Item Total Correlation**  (N=659) | **Corrected Item Total Correlation Strength^a^** |
| --- | --- | --- | --- |
| Evaluation | .631 | .666 | Very Good |
| Treatment | .691 | .652 | Very Good |
| Communication | 547 | .606 | Very Good |
| Professional Behavior | .248 | .227 | Low |

Abbreviation: SCIPAI = Student & Clinical Instructor Performance Assessment

^a^Criterion values as detailed in Raharjanti NW, Wiguna T, Purwadianto A, et al. Translation,

validity and reliability of decision style scale in forensic psychiatric setting in Indonesia.

Heliyon. 2022; 8(7):e09810. doi: 10.1016/j.heliyon.2022.e09810
